# Towards a standardized bioinformatics infrastructure for *N*- and *O*-glycomics

**DOI:** 10.1038/s41467-019-11131-x

**Published:** 2019-07-22

**Authors:** Miguel A. Rojas-Macias, Julien Mariethoz, Peter Andersson, Chunsheng Jin, Vignesh Venkatakrishnan, Nobuyuki P. Aoki, Daisuke Shinmachi, Christopher Ashwood, Katarina Madunic, Tao Zhang, Rebecca L. Miller, Oliver Horlacher, Weston B. Struwe, Yu Watanabe, Shujiro Okuda, Fredrik Levander, Daniel Kolarich, Pauline M. Rudd, Manfred Wuhrer, Carsten Kettner, Nicolle H. Packer, Kiyoko F. Aoki-Kinoshita, Frédérique Lisacek, Niclas G. Karlsson

**Affiliations:** 1 Department of Medical Biochemistry and Cell Biology, Institute of Biomedicine, Sahlgrenska Academy, University of Gothenburg, Gothenburg, 40530 Sweden; 2 Proteome Informatics Group, SIB Swiss Institute of Bioinformatics, Geneva, 1211 Switzerland; 3 Computer Science Department, University of Geneva, Geneva, 1227 Switzerland; 4 Soka University, Hachioji, 192-8577 Tokyo, Japan; 5 SparqLite LLC., Hachioji, 192-0032 Tokyo, Japan; 6 Department of Molecular Sciences, Macquarie University, Sydney, 2109 Australia; 7 Department of Biochemistry, Medical College of Wisconsin, Milwaukee, WI 53226 USA; 8 Leiden University Medical Center, Leiden, 2333ZA Netherlands; 9 Copenhagen Centre for Glycomics, Department of Cellular and Molecular Medicine, University of Copenhagen, København, DK-2200 Denmark; 10 Department of Chemistry, Chemistry Research Laboratory, University of Oxford, Oxford, OX1 3TA UK; 11 Graduate School of Medical and Dental Sciences, Niigata University, 950-2181 Niigata, Japan; 12 National Bioinformatics Infrastructure Sweden, Science for Life Laboratory, Department of Immunotechnology, Lund University, Lund, 22387 Sweden; 13 Institute for Glycomics, Gold Coast Campus, Griffith University, Gold Coast, QLD QLD 4222 Australia; 14 ARC Centre for Nanoscale BioPhotonics, Macquarie University and Griffith University, North Ryde and Gold Coast, NSW and QLD NSW 2109 and QLD 4222 Australia; 15 Bioprocessing Technology Institute, AStar, Singapore, 138668 Singapore; 16 Beilstein-Institut, Frankfurt am Main, 60487 Germany; 17 Section of Biology, University of Geneva, Geneva, 1211 Switzerland

**Keywords:** Glycomics, Mass spectrometry, Databases, Data publication and archiving

## Abstract

The mass spectrometry (MS)-based analysis of free polysaccharides and glycans released from proteins, lipids and proteoglycans increasingly relies on databases and software. Here, we review progress in the bioinformatics analysis of protein-released *N*- and *O*-linked glycans (*N*- and *O*-glycomics) and propose an e-infrastructure to overcome current deficits in data and experimental transparency. This workflow enables the standardized submission of MS-based glycomics information into the public repository UniCarb-DR. It implements the MIRAGE (Minimum Requirement for A Glycomics Experiment) reporting guidelines, storage of unprocessed MS data in the GlycoPOST repository and glycan structure registration using the GlyTouCan registry, thereby supporting the development and extension of a glycan structure knowledgebase.

## Abstract (web summary)

Glycomics is gaining momentum in basic, translational and clinical research. Here, the authors review current reporting standards and analysis tools for mass-spectrometry-based glycomics, and propose an e-infrastructure for standardized reporting and online deposition of glycomics data.

## Introduction

Posttranslational modifications of proteins play an essential role in modifying amino acids in proteins, thereby extending their functions and regulating their activities. A census of all possible protein forms, now commonly called proteoforms, was recently estimated^1^. In this renewed view of protein diversity, glycoforms are increasingly being shown to play a major role in both health and disease^2–5^. In fact, glycosylation has been shown to be involved in the vast majority of cellular interactions and complex networks. Glycosylation is the basis of most biological events, including protein structural stability, recognition, immunological responses, cancer metastasis and the attachment of pathogens to host cells as the first step in the process of infection^6,7^. Furthermore, the importance of glycosylation is highlighted by the extreme consequences of genetic defects in the glycosylation machinery^8^. Congenital Disorders of Glycosylation are a result of the loss of function of different enzymes involved in *N*-linked and *O*-linked oligosaccharide biosynthesis^9^, resulting in severe illness, organ failure and premature death. The importance of protein glycosylation demands that technologies used for structural determination and function are accurate, robust, and information-rich.

Here, we review the latest mass spectrometry (MS) technology for analysing released *N-* and *O-*linked glycans. We also describe the progress that has been made in glycobioinformatics software as well as structural/experimental databases and repositories. As researchers are requested to submit an increasing amount of analytical data into public repositories, we propose a standardized workflow for MS glycomics data recording based on community reporting guidelines and uploading of structural and experimental data to tailor-made databases and repositories.

## Methods and reporting standards for MS-based glycomics

### Structural characterisation of glycans by MS

At a first glance, MS is not the ideal choice for structural characterisation of glycans. While a precursor mass is sufficient to assign a composition (e.g. the number of constituting hexoses, *N*-acetylhexosamines etc.), it will not allow distinguishing between different isomeric structures, which is one of the major obstacles in the characterisation of glycans. A single mass measurement cannot resolve different isomeric monosaccharides such as glucose, mannose or galactose, nor does MS allow the assignment of pyranose, furanose or linear forms, or differentiation of enantiomers (D or L form).

More detailed insights into glycan structures can be obtained through MS/MS experiments, whereby the glycans are fragmented in the mass spectrometer. MS/MS CID (collision induced dissociation) and HCD (higher-energy collisional dissociation) fragmentation can help to determine, the primary sequence of a glycan, including branching points and elongation. However, additional input is required to identify the glycan linkage position (e.g., 1, 2, 3, 4 and 6) and configuration (α or β). Knowledge about the principles and rules of glycan biosynthesis—gained from structural gycobiology work using for instance NMR and studies characterising glycosyltransferase specificity—can reduce the number of conceivable MS assignments. In addition, specific cross-ring fragmentation in CID and HCD can sometimes enable linkage position assignment^10,11^. However, to fully assign a novel structure, a combination of MS, biosynthetic rules, chemical and enzymatic treatment, monosaccharide analysis, retention time and/or NMR is necessary. Multistage MS^n^ fragmentation^12^, ion mobility MS^13^ and ion spectroscopy^14,15^ can also be used to reduce the number of conceivable MS assignments. Furthermore, electron activation fragmentation techniques (referred to as ExD techniques)^16^ such as electron capture dissociation (ECD), electron transfer dissociation (ETD), electronic excitation dissociation (EED) and electron detachment dissociation (EDD) have been shown to provide extensive cross ring fragmentation allowing more detailed structural characterisation of glycans.

**Fig. 1:**
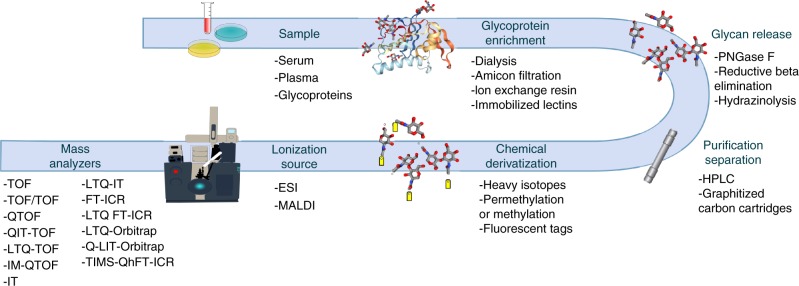
General Overview of a glycomics MS workflow. The analytical options at each step are shown below. A similar workflow has been described in ref. ^19^. TOF time of flight mass analyzer, TOF/TOF TOF tandem MS, Q quadrupole mass analyzer, QTOF tandem MS combining Q and TOF, IT ion trap mass analyzer, QIT dual operational Q and IT MS, QIT-TOF QIT combined with TOF tandem MS; LTQ(-IT), linear trap quadrupole mass analyser (a linear ion trap), LTQ-TOF tandem MS combining LTQ and TOF, IM ion mobility, IM-QTOF ion mobility cell/QTOF mass spectrometer, FT-ICR Fourier-transform ion cyclotron resonance mass analyzer, LTQ FT-ICR tandem MS combining LTQ and FT-ICR, LTQ-Orbitrap tandem MS combining LTQ and Orbitrap, LIT linear ion trap, Qh quadrupole/hexapole interface, TIMS trapped ion mobility spectrometry

Despite its limitations, MS has become the central tool for the study of protein glycosylation, largely due to its speed, high sensitivity, partial structural identification and capacity to deal with mixtures, and has been used extensively for glycomic screening/profiling^17,18^. The glycomic profiling of free and/or released glycans by MS has involved the use of a considerable variety of upfront dedicated isolation, derivatization and characterisation techniques that, together with increasingly sophisticated MS instrumentation, has been used to increase speed, depth and efficiency of analysis. A generic glycomic workflow for *N*- and *O*-glycans released from proteins has been described before^19^ and is summarized in Fig. 1.

It is important to point out that this review is focused on protein-based glycomics - which is different from glycoproteomics. While glycomics can generate detailed information about glycan structure(s), the methods used to release the glycans from the protein inevitably obliterate the localisation of the glycosylation site within a protein/peptide sequence. Glycoproteomics on the other hand tries to address glycosylation by analysing intact glyco-peptides/proteins. The caveat is that traditional fragmentation used on (glyco)peptides such as CID and HCD only provide limited information about oligosaccharide structure, and even site localisation can sometimes be difficult due to loss of the entire glycan^20^. In this context, ECD and later on ETD showed to be the fragmentation methods of choice for site localisation of glycans, since the fragmentation occurs primarily in the peptide chain^20,21^. Glycoproteomics analysis, at this stage, therefore can identify the site and mass (composition) of the glycan(s) on a particular site, but provides little details about glycan sequence, branching or linkage.

### Comparative glycomics

The goal of a glycomic experiment is not always to fully characterise all glycan structures in a sample. Instead, glycomic profiling is often applied to compare samples and focuses on the identification of abnormalities and differences. Several approaches such as MS, HPLC, LC-MS and capillary electrophoresis are used for glycomic profiling. They provide different levels of glycan characterisation ranging from mass profiling, to partial sequence and full structural assignments (based on complementary information), to absolute or relative quantification of individual structures in a biological sample.

In label-free MS-based analyses, the abundances of pseudomolecular ions (e.g. [M – *n*H]^*n−*^ or [M + *n*Na]^*n+*^ ions) are used to identify differences between samples. Furthermore, several quantitative glycomics methods are based on derivatization approaches and heavy labelled isotopes (reviewed in refs. ^22–24^). Quantification using stable isotope standards and MS has been shown to provide excellent precision in glycomics^25–27^ and glycoproteomics^28^. However, the low number of freely available stable isotope standards is currently the limiting factor for implementing absolute quantitation in MS-based glycomics for a wider range of glycans available from single cells or tissues.

Relative quantification using fluorescent tagging in connection with HPLC or capillary electrophoresis provides the benefit of stoichiometric response from individual glyco components and is the gold standard for glycomic relative quantification^29^. Cross-laboratory comparisons have shown that MS can provide similar quantification results^30,31^, where differences between laboratories can mainly be attributed to differences in sample preparation and data accumulation protocols. This illustrates the need to accurately record protocols for structural assignment of a well-defined sample data and sample handling protocols, and quantitative aspect.

### Adopting omics reporting guidelines for glycomics

MS based omics entails analysing a multitude of samples generating large amounts of data, and using software to transform these data into biological information. To make this process transparent and reproducible, there is a need for consistent reporting of experimental methods and procedures in publications. Many omics fields have addressed these concerns by developing guidelines for the reporting, collecting and distributing of data and information. This started with MIAME launched for the handling of microarrays^32^, followed by the MIAPE guidelines for proteomics^33^, STRENDA in enzymology^34,35^, CIMR in metabolomics^36,37^ among others. There are currently more than 150 reporting guidelines published and registered in the FAIRSharing portal^38^.

As was discussed in the previous paragraph, structural characterisation of glycans using only MS is difficult. Multiple guidelines are required for the multiple techniques that are used to convert the analytical data into detailed structural glycomic information. To acknowledge the complexity of glycan structural characterisation, the glycomics community launched the MIRAGE (Minimum Information Required for A Glycomics Experiment) initiative in 2011. The MIRAGE initiative is formed by experts from the diverse areas of glycomics research and supported by the Beilstein-Institut^39^. Up to now this has resulted in guidelines for glycomics sample preparation^40^ (10.3762/mirage.1), MS analysis^41^ (10.3762/mirage.2), glycan microarray analysis^42^ (10.3762/mirage.3), and liquid chromatography analysis^43^ (10.3762/mirage.4). The MS guidelines require not only reporting of experimental conditions, but also disclosure of raw MS data and annotated spectra. Making these guidelines widely applicable require the development of workflows that describe what is to be reported and how to record glycomics MS data. In addition, adoptability of the guidelines requires a web-based software pipeline that facilitates the flow from MS data acquisition to public disclosure of raw data and reporting of data structural interpretation/annotation.

## The current landscape of glycomic e-infrastructures

### Glycan structure repositories

In 2008, the NIH work group “Frontiers in glycomics” emphasised the need for a curated, sustainably funded glyco-structure database^44^. Accounting for the variety of analytical methods used to assign glycan structures, the proposed structural database was expected to contain associated information about experimental and biosynthetic data. Pioneering attempts to create a comprehensive glycomic database were made in the 1980s with Carbank^45^. The Carbank institutors also implemented regular updates with information from new publications. Unfortunately, a funding crisis stopped this effort in the 1990s and the project was discontinued, but the assembled data lived on in the next-generation databases including SWEET-DB^46^ and GlycosuiteDB^47^ (later incorporated into UniCarbKB^48^ and GlyConnect^49^, both having the same agenda as their ancestor. Then, integrative initiatives arose with the goal of centralising scattered data (e.g. GlycomeDB^50^), as well as combining it with in silico analytical tools such as GLYCOSCIENCES.de^51^, KEGG GLYCAN^52,53^ and repositories provided by the Consortium for Functional Glycomics (CFG, http://www.functionalglycomics.org/fg/). The progress in the field was somewhat chaotic at the turn of the century, but has significantly evolved lately through a new generation of centralised and integrative resources, each one located on a different continent and being developed in mutual recognition. These are GlyGen in the US (http://www.glygen.org/), Glycomics@ExPASy^54^ (https://www.expasy.org/glycomics) in Europe and GlyCosmos in Japan (https://glycosmos.org/). This recent trend may finally provide a long-term solution for stable and financially supported resources for glycobiology. For instance, GlyCosmos includes GlyTouCan (https://glytoucan.org/)^55^, a registry that provides glycan structures with unique identifiers. GlyTouCan provides a foundation for developing complementary repositories, where each unique glycan recorded can be associated with additional experimental information, such as MS data, HPLC retention times and NMR spectra.

### Bioinformatic resources for MS-based glycomics

To capture information contained in glycomics MS/MS data, UniCarb-DB was launched in 2011^56,57^. Since its introduction, several versions of UniCarb-DB have been released, mainly to improve the glycomics data quality, to increase the number of entries and to advance the usability of the application. UniCarb-DB is currently integrated in Glycomics@ExPASy and provides the framework for accessing experimental MS data that comprise fragmentation spectra, associated structures and metadata about biological origin. Currently, UniCarb-DB contains structural and fragmentation data of *O*-glycans and *N*-glycans obtained in positive and negative MS ion modes. Additional MS fragmentation spectra of glycans are provided in the NIST Glycan Mass Spectral Reference Library (https://chemdata.nist.gov/glycan/spectra)^58^.

In parallel to the expansion of glycan structure databases, there has been slow but steady progress in the development of software for glycomics data analysis. The early GlycosidIQ automated the comparison of observed fragments with theoretical glyco-fragments derived from a structural database^59^. This approach has been adopted in commercial software^60^. GlycoReSoft was developed to aid glycan detection from LC-MS runs to compare different samples^61^. Other approaches convert mass spectra into structures relying on spectral libraries^57,62^. More advanced tools for glycomics analysis use partial de novo sequencing^63^ including GlycoDeNovo^64^ and the recently published Glycoforest^65^. High-throughput glycomics MS annotation tools (GRITS Toolbox^66^, www.grits-toolbox.org/) and quantitation tools^67^ are now available and increase the need for a common data exchange format. Providing data in an agreed format will help to make data publicly accessible, so that they can be scrutinized by others and used for the validation and curation of glycan structures, for instance those deposited in the GlyTouCan registry.

## A mirage-compatible e-infrastructure for MS-based glycomics

In order to implement the MIRAGE guidelines^39^ into an MS-based glycomics e-infrastructure, two existing guidelines were used; (1) glycomic sample preparation^40^ and (2) defined MS conditions^41^. The curators also proposed a HPLC experimental module, expanding on the guidelines to enable recording of LC-MS parameters. This section is planned to be expanded since the MIRAGE LC-guidelines recently were published^43^.

This first version of a data recording workflow will focus efforts on the most essential implementation of qualitative, structural information. Quantification guidelines will only be addressed at a superficial level, with expected expansion in subsequent versions. This can be justified considering that workflows for quantitative glycomics are still evolving and that the basic level of methods and software tools is yet to become common practice. Due to the lack of a long-term global public repository for MS glycomics raw data, the requirement to provide this quantitative information as part of the submission has not yet set to be compulsory.

**Fig. 2:**
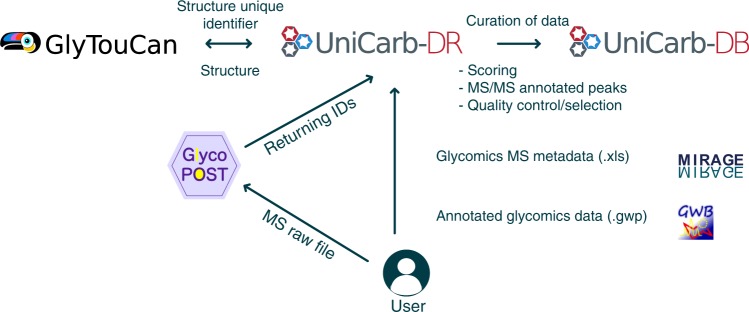
Workflow for MIRAGE data submission to UniCarb-DR. The workflow was designed to support the MIRAGE guidelines for sample preparation, including HPLC, MS and annotation. Metadata that are relevant to the entire experiment can be manually recorded in the on-line form accessible on the UniCarb-DR website (http://unicarb-dr.biomedicine.gu.se/) that automatically generates spreadsheets (.xls). GlycoWorkBench files (.gwp) are proposed to be used for data annotation of individual structures. MS raw files can be submitted to GlycoPOST and returned IDs can be included in the MIRAGE report. UniCarb-DR also communicates with the global glycostructure repository GlyTouCan to generate unique identifiers for structures submitted to UniCarb-DR. UniCarb-DR will become one source of curated data in UniCarb-DB

To support an e-workflow, we created the data repository UniCarb-DR (http://unicarb-dr.biomedicine.gu.se/) to facilitates submission of glycomics MS^n^ data in compliance with the MIRAGE guidelines as part of a publication submission process (Fig. 2). This repository will serve as the interim storage of experimental MS fragment data and structures before data curation and annotation and subsequent transition into the UniCarb-DB database. An author can browse and re-enter submitted data before it is uploaded to the UniCarb-DB repository. We assume that in the near future journals will require data submission to be compulsory prior to publication as for other omics data. Hence, the user can submit the data, referring to it as a “manuscript”. For data uploaded after publication, PubMed ID (PMID), available from https://www.ncbi.nlm.nih.gov/pubmed/ can be included.

Data deposition in the repository first requires user registration and login at http://unicarb-dr.biomedicine.gu.se/signup. Next, the user must provide a number of files and information (Fig. 2) including:

1. A compiled file with MIRAGE data (see example spreadsheet in Supplementary Data 1).
2. Compiled information about structures (proposed format is GlycoWorkbench)^68^.
3. Location of publicly accessible unprocessed MS files.
4. Unique structure identifier (this information is automatically generated by communication between UniCarb-DR and the GlyTouCan structural repository^55^).

Further information about these four steps is provided below. A detailed protocol for how to fill in spreadsheets, GlycoWorkbench files and how submit data to UniCarb-DR is available in Supplementary Note 1.

### Step 1: Recording MIRAGE data using webform

Experimental data needs to be provided in a spreadsheet with data fields reflecting the general structure of the MIRAGE guidelines. Prefilling and downloading of the MIRAGE compliant spreadsheets are possible in the web form (http://unicarb-dr.biomedicine.gu.se/generate). Three different spreadsheets are available: (1) sample preparation, (2) LC and (3) MS guidelines. These can be generated individually or combined into one file containing several sheets (see Supplementary Data 1). These spreadsheets can be modified off-line using common software packages such as Excel. The templates use controlled vocabularies (e.g. for tissue, taxonomy or instrument description) to facilitate user input and simplify data exchange. They can be extended for harmonisation with other standard initiatives such as the HUPO Proteomics Standards Initiative (http://www.psidev.info). Additional glyco-related ontologies are proposed (Supplemenary Data 2) and will be expanded in line with existing ontologies proposed by the MIRAGE commission and subsequently included in the input form.

### Step 2: Recording structures and MS fragmentation

The open source software GlycoWorkbench developed within the EuroCarb project to assist manual annotation of MS/MS data^68^ can be used to record glycan structures. GlycoWorkbench provides a straightforward interface to draw glycan structures in cartoon formats using the embedded GlycanBuilder module^68^. Glycan structures are stored in a linear format (.gws) for easy parsing and recording into databases. All recorded data can be stored in an XML-type Glycoworkbench file (.gwp file extension) (Supplementary Fig. 1). A Glycoworkbench template is available at https://unicarb-dr.biomedicine.gu.se/generate and an example of a filled in file is available in Supplementary Data 3. GlycoWorkbench allows the recording of individual structures as a “Scan” with associated fragment data (fragment list is imported from MS software as centroided data). We suggest utilizing the ability of GlycoWorkbench to record ion trees, using Glycoworkbench “Scans” to record MS^2^ (i.e. MS/MS) for each structure, and sub-“Scans” to record MS^3^, MS^4^ etc. A GlycoWorkbench file that includes several structures and MS^n^ data, for each structure “Scans” and sub-“Scans” needs to be defined directly under the “Workspace” item.

Supplementary Fig. 1 shows the sections that are typically included in a.gwp file. A tag is represented by the “<” and “>” symbols and defines the different elements in a file. These elements are delimited by a start tag e.g. <scan> and an end tag, e.g. </scan>. The example shown in Supplementary Fig. 1 belongs to a single structure somewhat simplified, highlighting important MIRAGE tags. In order to be MIRAGE-compatible, we introduced a “Notes” section for recording orthogonal assignment methods, scoring and validation (see “MIRAGE parameters in UniCarb-DR” below). The format of the “Notes” section needs to be respected in order to upload its content to UniCarb-DR (see Supplementary Methods for more details on the proposed “Notes” format).

### Step 3: Depositing MS raw files

To host the vast volume of glycomics MS raw data, we propose a model of data sharing similar to the one implemented in proteomics by the ProteomeXchange consortium^69^ that includes PRIDE^70^ and JPOST (Japan ProteOme STandard Repository/Database), among others. To provide open data access that complies with the MIRAGE requirements, we engaged with JPOST. We developed a pipeline enabling permanent data storage in GlycoPOST (http://glycopost.glycosmos.org/), a dedicated repository for MS-based glycomics data. The current model requires submission of MS raw data to GlycoPOST, whilst MIRAGE and GlycoWorkbench files are uploaded and read by the UniCarb-DR submission workflow. To further simplify this process, we are working on integrating submission of raw data, annotated spectra and MIRAGE meta-data in a seamless workflow using both UniCarb-DR and GlycoPOST (Fig. 2). Meanwhile, the applications are streamlined since the same MIRAGE spreadsheets are used in both applications and the user has the option of including GlycoPOST generated URLs of raw data into MIRAGE spreadsheets before submission to UniCarb-DR. GlycoPOST also accepts and stores other types of files, allowing users to upload information about experimental design, sample log files including quality control samples and blank runs, as well as additional information that is potentially useful for checking the quality and reproducibility of glycomic experiments containing multiple samples. Hence, data repositories and the MIRAGE commission will depend on each other when the next version of guidelines is to be developed. Alternatives to GlycoPost for the long-term storage of MS raw data can potentially be included in the workflow. For instance, we have also used an MS Laboratory Information Management System called Proteios Software Environment (http://www.proteios.org)^71^ to upload data to Swestore (http://www.snic.se/allocations/swestore/), where Swestore generated Uniform Resource Identifiers (URIs) that have been included as part of scientific publications^72^. In this case, MS raw data are provided both as vendors’ preferred format and as files converted into the open source mzML format^73^. The mzML format is not only describing spectral data but also contains information requested in the MIRAGE guidelines. In the short term, we strongly recommend uploading mzML data separately to GlycoPOST or other repositories for non-vendor software dependent data access. In the long term, this submission of raw MS data can be integrated in the UniCarb-DR upload. We note that the MIRAGE bioinformatics subgroup has considered mzIdentML^74^ and mzTab^75^ potential formats that could be augmented with glycomics data, and this will also be implemented in the long term.

### Step 4: Registration of submitted glycan structures

GlyTouCan^55^ is a glycan structure repository promoted by the glyco-community as the prime location for generating unique identifiers for individually reported glycan structures and compositions. Glycan structures should be submitted to this repository as part of the publication of glycomics data. To avoid duplicate submissions to both UniCarb-DR and GlyTouCan we have developed a tool that assesses whether the structures submitted to UniCarb-DR are already deposited in GlyTouCan. In this case, the GlyTouCan ID provides a link to UniCarb-DR. If a UniCarb-DR submitted structure is not available in GlyTouCan, a new ID will be generated and communicated to UniCarb-DR. This process will commence after the submission of data to UniCarb-DR.

### MIRAGE parameters in UniCarb-DR

The MIRAGE guidelines are generic and flexible in order to collect information from different types of experiments studying glycoconjugates. However, the use of commonly defined vocabularies is required to compare data within UniCarb-DR and to share data with other glycomics and life science databases. To preserve the flexibility of the MIRAGE guidelines in the reporting process we propose free text fields to describe experiments, whilst a rigorous reporting language is implemented only for key MIRAGE parameters (e.g., tissue, MS device). Inspired by the organization of PRIDE^76^, four different types of formats of the MIRAGE parameters were encoded in UniCarb-DR (Table 1) and outlined in the Supplementary Methods and Supplementary Data 2. To comply with the controlled vocabulary but still enable glossary update, new terms can be suggested by sending a request to administrators of UniCarb-DR at http://unicarb-dr.biomedicine.gu.se/about.

**Table 1:** Examples of parameters identified and included in the MIRAGE guidelines

Defined formats	Existing ontologies	New parameters in glycoanalytics	Free text
Unique identifiers for structure and entry	Cell lines www.clo-ontology.org/	Treatments and orthogonal methods for isolation/characterisation	Growth/harvest conditions for recombinantly produced material
Dates	Species www.ncbi.nlm.nih.gov/taxonomy	Software used for data processing and structure assignment	Treatments and/or storage conditions for material isolated from tissues
Oligosaccharide structure (.gws, glyco-CT^84^ and WURCS^85^)	Tissues meshb.nlm.nih.gov/record/ui?ui=D014024	MS scoring method	Synthesis steps for chemically derived material
	MS devices www.ebi.ac.uk/ols/ontologies/ms	Validation of assigned structure	Purification steps
	Software www.ebi.ac.uk/ols/ontologies/ms		Sprayer features
	Attached proteins www.uniprot.org/		Voltages and other parameters relevant for ESI experiments
			CID/HCD settings

The columns describe (from left to right): defined formats to control the input and presentation of certain data, ontologies or existing vocabulary that can be adopted from various sources, parameters that need to be expanded into ontologies or controlled vocabulary to use in glycomics, free unrestricted text

### Upload of MIRAGE compatible MS/MS spectra to UniCarb-DR

**Fig. 3:**
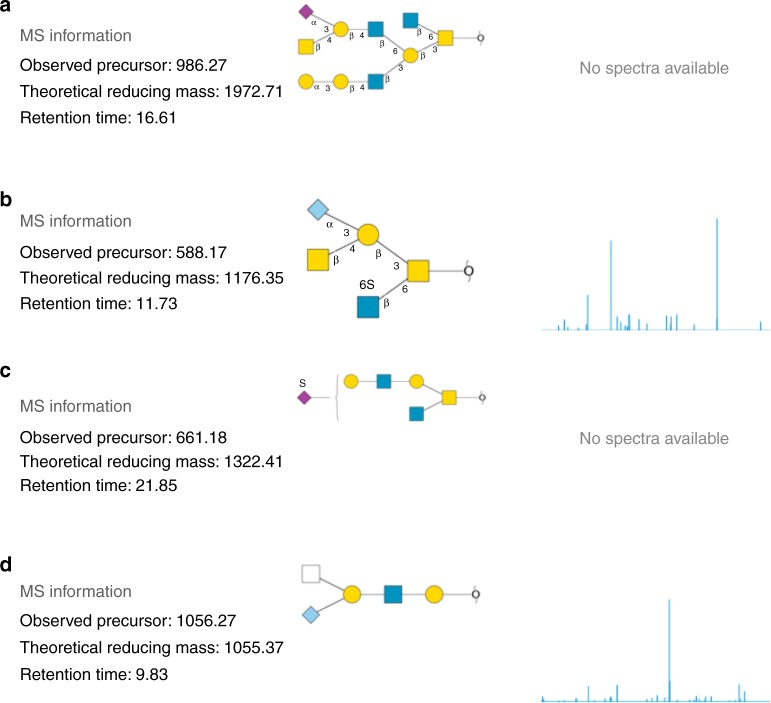
Different types of entries managed by the UniCarb-DR submission tool. The glycan structures are displayed in SNFG cartoon notation^83^. **a** Structure proposed from LC-MS without an LC-MS/MS spectrum. **b** Fully assigned structure with an associated LC-MS/MS spectrum. **c** Partially assigned structure proposed from LC-MS without an LC-MS/MS spectrum. **d** Partially assigned structure with an associated LC- MS/MS spectrum

MIRAGE-compliant data sets along with data stored in.gwp files of both individual intact structures and fragmentation spectra can be submitted as supplementary material associated with a publication. We also propose uploading these collected and structured glycomic information (spreadsheets and.gwp files) to http://unicarb-dr.biomedicine.gu.se/uploadData. Before uploading, the user is required to register at http://unicarb-dr.biomedicine.gu.se/signup. The database allows structures of full and partial assignment to be uploaded (Fig. 3). The reporting of orthogonal methods (i.e. NMR, HPLC retention time mapping, and chemical/enzymatic treatment) is also possible and justifies the fact that UniCarb-DR can be used to accept structures where MS, but not MS^n^ data, has been collected. Figure 3, displays examples of assigned structures in UniCarb-DR (http://unicarb-dr.biomedicine.gu.se/references/1), both with and without associated fragmentation data. In the latter case, structures were assigned based on retention time (RT) and biosynthetic knowledge about the constituting monosaccharides, linkage position and configuration. Observed RT is of course of limited use outside a particular experiment. Hence, we promote recording relative RT using external RT markers that generate, for instance, a nominal size corresponding to number of glucose units (GU)^77^ or relative to an internal common landmark oligosaccharide^78^.

We have assembled an expandable list of treatments and orthogonal methods commonly used for isolation/characterisation in glycomics experiments (Table 1 and Supplementary Data 2). The current records in UniCarb-DR have been uploaded using data generated in various laboratories by researchers in the author list. During this process we found that the requirement to record the full information about individual structures (e.g. scoring and orthogonal method validation) is time consuming due to lack of software, and is often not feasible. Hence, UniCarb-DR is also accepting data with partial MIRAGE records for an individual structure, requiring only the record of the precursor ion mass but no information about scoring and validation. Many authors of this paper are part of the MIRAGE committee. In light of these limitations, we have proposed to only require partial MIRAGE compliance for submitted records of an individual structure, with the commitment to move towards full MIRAGE compliance as glycomics software develops further.

## Discussion and future perspectives

The lack of an established formalised description of glycomics experiments may stall progress in the glycobiology field. The research community has always relied on sharing scientific results. Here, we are proposing a solution for sharing glycomics structures with associated MS experimental conditions and data. For this we are using spreadsheets in combination with GlycoWorkbench files. This format is a step towards enforcing MIRAGE-compliant scientific publications in glycomics. Past experience in introducing guidelines for glycomics studies as part of publications^79^ has shown that, if there is a clear pathway and format, researchers will conform to get their manuscripts published in quality journals. With the tools presented in this report, glycomics MS/MS^n^ reporting standards can be adopted at an early stage of a project. The spreadsheet can be completed and modified as the project unfolds and the use of GlycoWorkbench files for saving glycomic structural interpretations can be implemented for data housekeeping. Both the spreadsheet and the.gwp formats are flexible enough to support a variety of glycomics MS, requiring only limited modifications of templates provided. Hence, journal editors will be in the position to ensure MIRAGE compliance by requesting that authors provide these template files as supplementary data. The MIRAGE committee is continuously corresponding with relevant journal editors on novel MIRAGE protocols, software tools and repositories to implement MIRAGE guidelines as part of the publication requirements. With an increasing awareness of MIRAGE formats and associated enabling technologies, we expect that an increasing number of researchers and reviewers will insist that, not only their own, but also other groups’ data are MIRAGE-compliant in scientific publications.

The use of spreadsheets (generated form web form, see Supplementary Data 1), deposition of raw data (e.g. in GlycoPost) and the GlycoWorkbench.gwp format are  supporting the upload of glycomics MS metadata to UniCarb-DR. This workflow has been tested for datasets of intact as well as reducing-end-derivatized glycans, which were analysed by MS, LC-MS and -MS/MS in negative and positive ion modes using CID and HCD fragmentation. All the data can be assembled manually, allowing the workflow to be used by both beginners in glycomics as well as advanced glycomic MS institutes. However, another purpose of defining the upload format is to provide a template for the output from software-aided glycomic discovery pipelines. The GlycoWorkbench structure format has already been adopted in other glycomic commercial (GlycoQuest, Bruker, Bremen Germany) and academic (GRITS Toolbox (http://www.grits-toolbox.org/) software projects^66^. Hence, automated submission to UniCarb-DR is likely to be easily implemented for these tools.

The focus of this report is on *N*- and *O*-linked glycans released from proteins and their spectra generated by CID and HCD. This is because the vast majority of data currently available are *N*- and *O*-linked CID fragmentation data using positive and negative ion modes. UniCarb-DR will also be able to host CID/HCD data of free glycans, glycans released from glycolipids and glycosaminoglycans. In principle, the repository could also host data of intact glycolipids, but the formalisation of the aglycon is currently missing in GlycoWorkbench. The lack of detailed structural information about the glycan moiety in global glycoproteomics data complicates recording of these data with the workflow presented here. However, the main obstacle for including glycoproteomics data is that UniCarb-DR currently is not capturing peptide sequences and glycosite information, and is not associating data to a protein. For the time being, glycopeptide MS data is being collected in databases such as MS-Viewer^80^ and, as partially curated data, in GlyConnect^49^ where they are integrated with multiple related sources of information on the recorded attached glycan composition.

Other workflows utilized in glycomics, such as permethylation and other type of derivatisation recordable in Glycoworkbench followed by MS with or without coupled LC separation, are easily implemented if the spectra are from single isomers. With several isomers present in one spectrum, these data can still be recorded in GlycoWorkbench (several structures recorded in one “Scan”), but the UniCarb-DR format will need to be modified in future versions to enable easy upload of this mixed-structure data. Similar concerns apply to workflows involving multi-stage MS^n^, despite the flexibility of recording sub-“Scans” in GlycoWorkbench. Glycomic MS workflows including ion mobility MS will require updating the spreadsheets and Glycoworkbench files with information about collisional cross sections and ion-mobility parameters currently not considered in MIRAGE or will need to link to databases that contain cross section information from carbohydrates such as GlycoMob^81^. Fragmentation data generated by ExD or other type of fragmentation techniques producing non-standard fragments cannot be recorded in GlycoWorkbench and will require further adjustments of the UniCarb-DR upload procedure. This would involve expanding the type and associated metadata for individual fragments. Currently, non-standard fragment peaklist can be uploaded, without annotation of non-standard fragments. We also request help from the community to identify additional major glycomics workflows for us to adapt the data submission accordingly.

In addition to adapting the submission process to a broader range of experimental workflows, we are also aiming to automate submission to UniCarb-DR. To this end, we plan to accept direct submissions from glycan structure assignment tools such as Glycoforest^65^. In 2020, a web version of Glycoforest will manage the automation of structure assignment to MS/MS spectra. Glycoforest first generates consensus spectra from the MS/MS data, and then assigns structures to the consensus spectra. The resulting assignments can be manually checked and if necessary corrected by the user. The direct submission of spectra and their associated assignments will contribute to the expansion of UniCarb-DR and help reduce human error in the submission process.

The commitment to store glycomics MS datasets is essential. The annotation of glycomics LC-MS data is currently based only on the knowledge of the interpreters^65^, be it a software or a human researcher or both. From this it can be concluded that it is highly unlikely that all information from a glycomic raw data set will be extracted in a single analysis. Hence, glycomic raw MS data should be considered as libraries that will be re-analysed to harvest new knowledge and to ask new questions. This is even more important if glycomics evolves similarly to proteomics and will increasingly rely on data independent acquisition^82^ in addition to data depend acquisition as a means to generate data from clinical or other reference samples. These glycomics libraries will provide essential information for hypothesis-driven glycomics. Similar to PRIDE and ProteomeXchange initiatives, the glycomics community needs to voice the unanimous opinion that this is needed, and target both national and international life science e-infrastructure organizations and journals. The MIRAGE committee already identified this requirement by introducing the recommendation for raw data deposition in the guidelines.

A pipeline for curation of experimental data from data repositories into databases is changing how curated structural databases will be generated. The previous top-down approach of a database generator and curator searching literature for information will shift to researchers submitting and managing their own data. Researchers and curators will need software tools to help in the curation process. The metadata in the reporting guidelines and evaluation of the accompanying publication, complemented with present and future biosynthetic knowledge, will aid the curation process. This process must remain objective and transparent in that information can only be added, but not deleted or altered (unless permitted by the data supplier). The MIRAGE guidelines can only be strengthened by such an approach that supports the unbiased assessment of data quality. The mission of UniCarb-DR and Unicarb-DB is to support the development of a knowledgebase of glycan structures by providing the pipeline for storage and curation of glycomic experimental MS data.

## Supplementary information

Supplementary Information

Description of Additional Supplementary Files

Supplementary Data 1

Supplementary Data 2

Supplemenary Data 3
