# Supplementary material for: Towards a standardized bioinformatics infrastructure for N- and O-glycomics
Source: Nat Commun. 2019 Jul 22;10:3275. doi: 10.1038/s41467-019-11131-x (PMC6796180; doi:10.1038/s41467-019-11131-x)
Supplement: Supplementary file 1 — Supplementary Information [file 41467_2019_11131_MOESM1_ESM.pdf]

Towards a standardized bioinformatics infrastructure for *N*- and *O*-glycomics

Rojas-Macias *et al.*

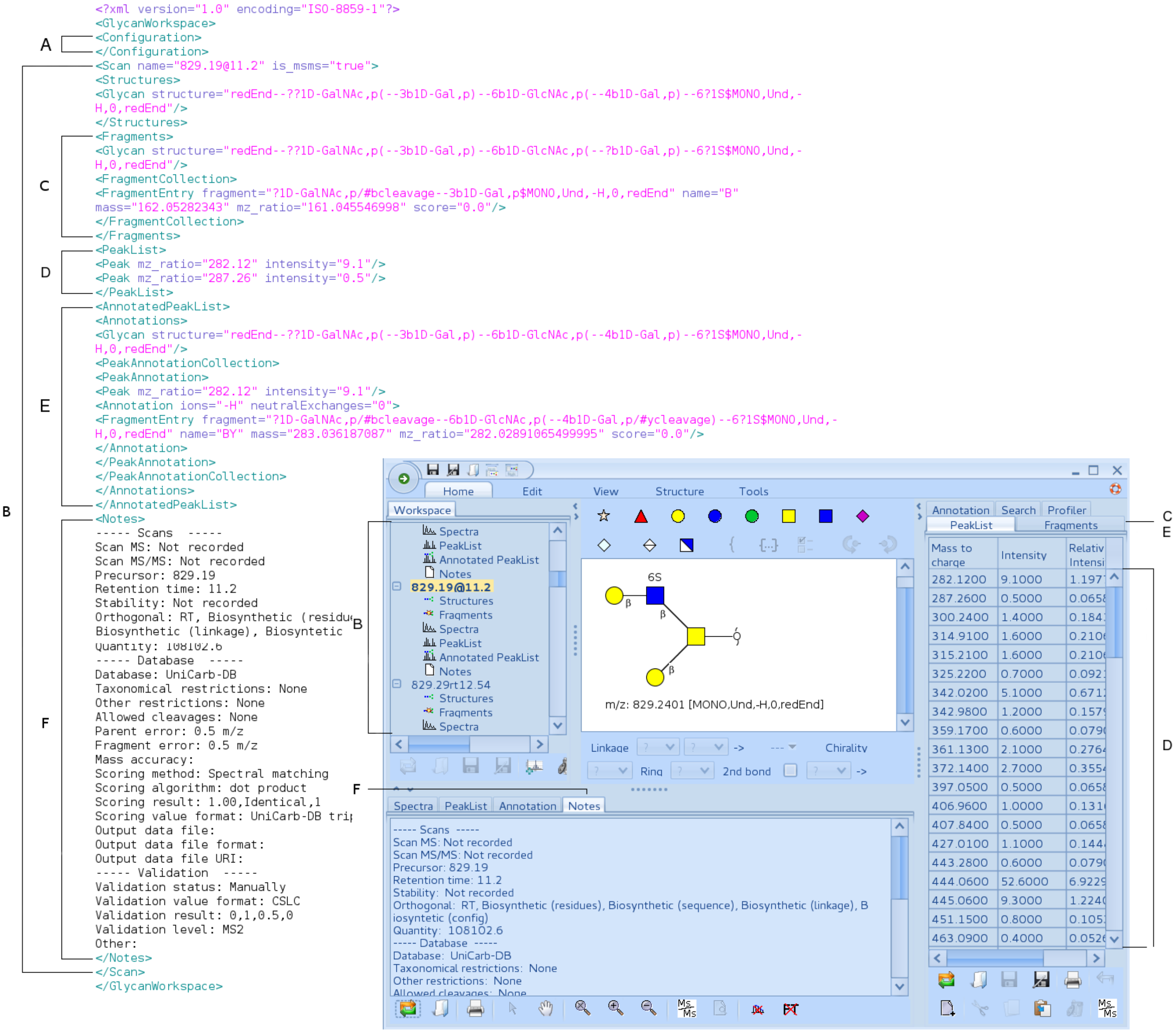

Supplementary Figure 1. Main sections in the GlycoWorkBench template. The graphical interface of GlycoWorkBench is divided into different panels. The central panel displays the glycan structure in SNFG cartoon notation. Below the structure, multiple panels can be accessed through their respective tabs. The last tab corresponds to the ‘Notes’ section (F). Here, additional MIRAGE data can be included. All structures in the file are listed in the panel on the left (B). In this case, the names correspond to their *m/z* values and retention times. The peak list (D) and the list of fragments (C and E) are included on the right side panel.

The above mentioned sections are shown as they are stored in the respective .gwp files. GWP files follow XML formatting rules where every element in the file is delimited by tags. The notes for example are defined between the <notes> and </notes> tags (F). Sections can be nested into other sections and can be present in multiple instances.

SUPPLEMENTARY NOTE 1: MIRAGE protocol

Filling in the MIRAGE web-form

This step is to generate Excel spreadsheet(s) compatible with MIRAGE guideline.

1. Go to website: http://unicarb-dr.biomedicine.gu.se/generate
2. The Mirage spreadsheet contains three parts. The users can choose individual or all spreadsheets fitting the experimental setting. Then click **NEXT**.

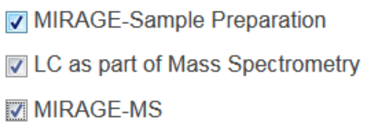

NOTE: Required fields are marked with an * (If the field is not relevant for the experiment, it can be filled in with a "NA"-Not applicable).

After filling in the selected part(s), an Excel file can be created after clicking Generate. The user can modify the content during the filling-in (website) or after downloaded Excel spreadsheet(s).

3. MIRAGE-Sample Preparation section

The user needs to fill in basic information especially those marked with *. If the cursor stays in column for a few second, a guideline will appears which will help the user to fill in the information. Or the user can select items from drop list if provided.

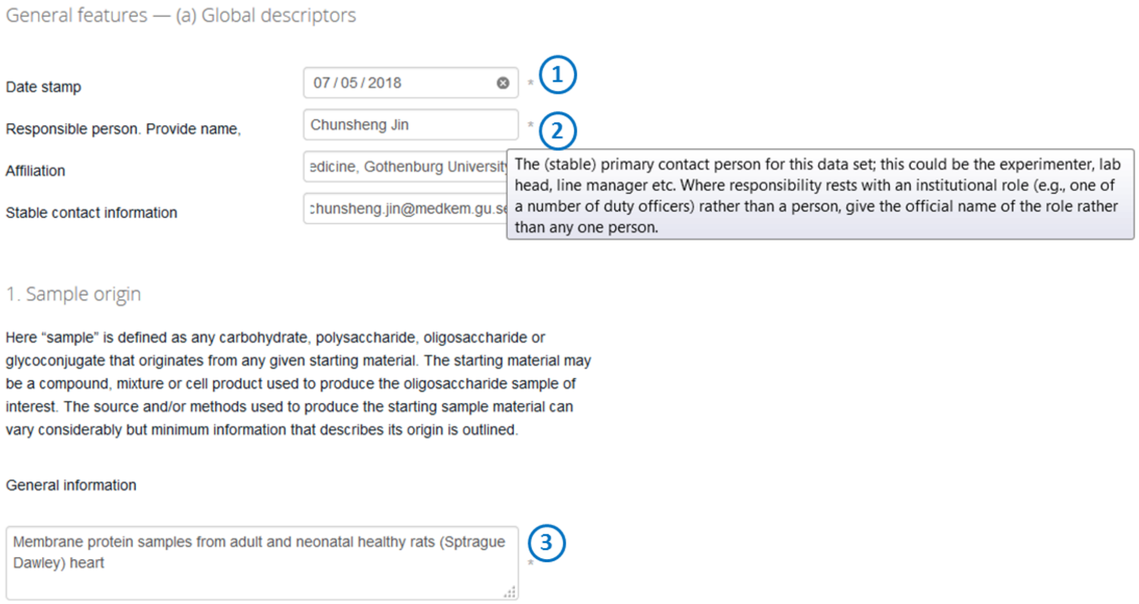

(1) The date on which the work described was completed; given in the standard 'YYYY-MM-DD' format (with hyphens).

(2) The (stable) primary contact person for this data set; this could be the experimenter, lab head, line manager etc. Where responsibility rests with an institutional role (e.g., one of a number of duty officers) rather than a person, give the official name of the role rather than any one person. In all cases give affiliation and stable contact information. This information can be made available as part of an authors' list or in an acknowledgment section

(3) Describe how original starting sample material was generated or where it was obtained. Starting material descriptions are further delineated by biologically or chemically derived material.

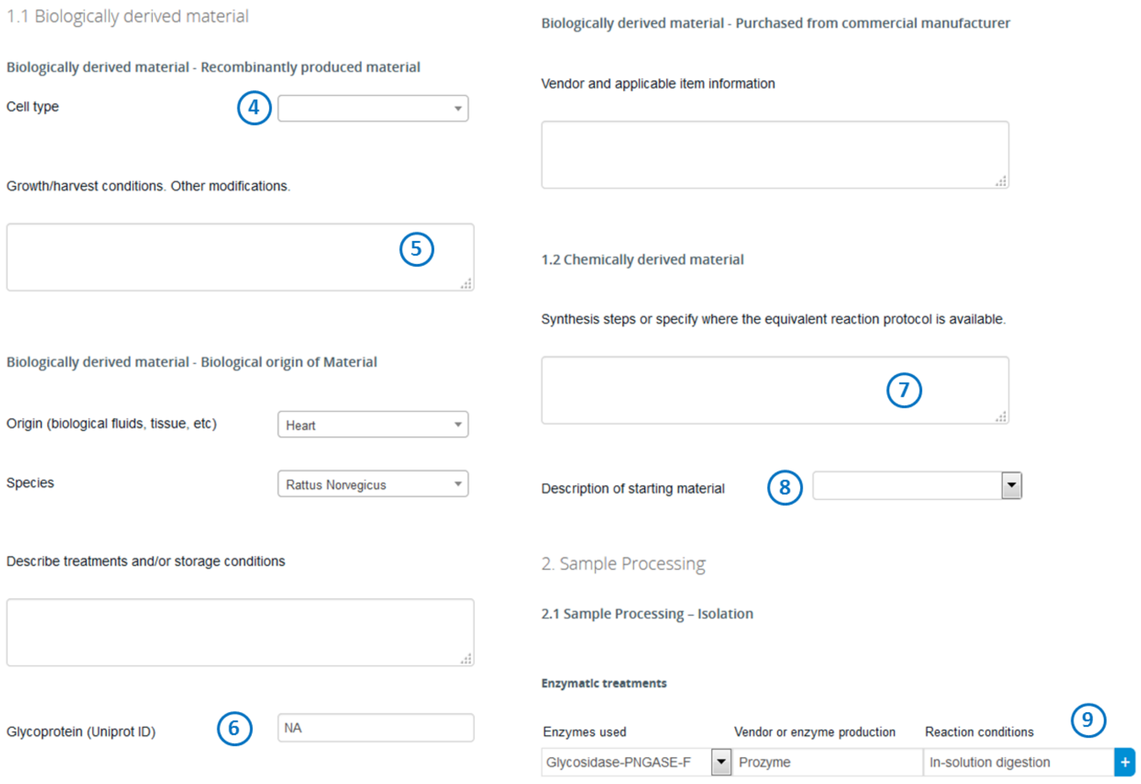

(4) Name of cell line (e.g., CHO, HEK, NS0 etc.)

(5) Growth/harvest conditions should be specified. Any modifications to cells that influence the characteristics of the starting material (e.g. genetic manipulations) should also be stated.

(6) Uniprot ID (e.g., PQ2771)

(7) If samples were synthetically derived, provide information.

(8) Define the type of starting material used or produced that contains the oligosaccharide to be used/analysed in subsequent experiments. These may include glycoprotein(s), proteoglycan, glycolipid, GPI-anchored, free-oligosaccharides, sugar-nucleotides or synthetically derived material but are not limited to these definitions.

(9) Processing may include methods to remove the oligosaccharides from the starting material prior to downstream experiments or conversely the starting material may also be altered so the oligosaccharide remains conjugated to non-carbohydrate material such as chemical (e.g. linker) or biological (e.g. peptides) components.

For enzymatic treatments, (i) describe any enzymes used to for the purpose of oligosaccharide removal (e.g. PNGase F) or for modification of the starting material (e.g. trypsin protease); (ii) specify where it was obtained (vender) or for enzymes produced in-house, describe expression and purification procedure; (iii) state if sample material was treated in-solution or immobilized (SDS-PAGE, PVDF etc.) as well as temperature, duration, volume, enzyme concentration.

For chemical treatments, it refers to the technique for oligosaccharide release or other chemical modifications (e.g., hydrazinolysis, β-elimination etc.). The reaction condition should contain temperature, duration, volume and chemical concentrations.

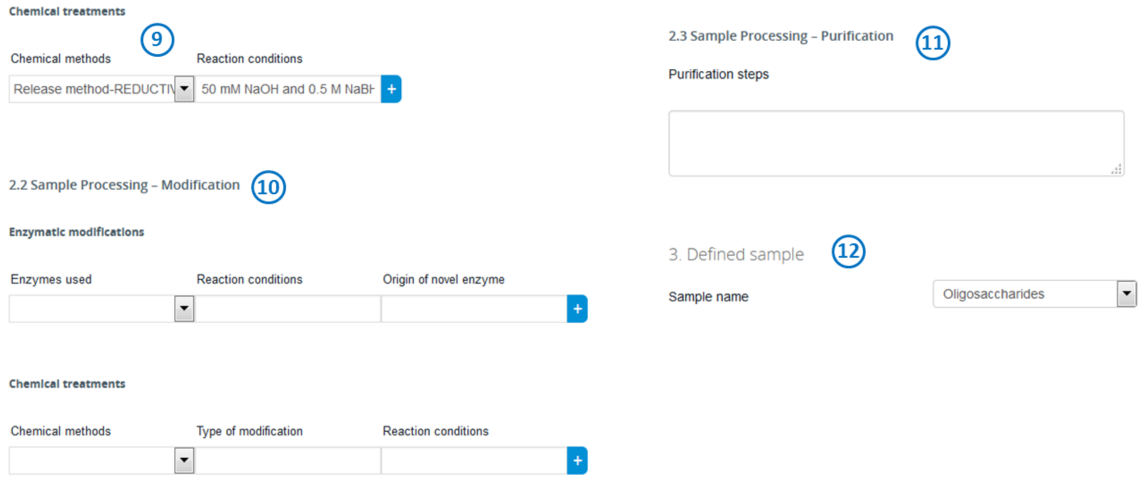

(10) Sample processing-Modification

For enzymatic modifications, (i) describe any treatments made to the isolated material; (ii) enzyme concentration, supplier, biological source, incubation time and temperature, (iii) if novel glycosidase was used, provide information indicating the origin (i.e. species) of the enzyme.

For chemical modifications, (i) describe any treatment made to the isolated material; (2) explain the type of modification employed (e.g., hydrolysis, sample tagging including fluorescent labels, isotopic labelling, permethylation/peracetylation, etc.); (3) source of materials, description of kits used, reaction conditions and detailed workflow.

(11) Sample processing-Purification

Specify all steps used to purify starting material after isolation/modification steps. Examples of procedures include solid phase extraction (SPE), liquid-liquid extraction or other chromatographic methods. For each method describe the all experimental materials (e.g., stationary phase) and methods (e.g., flow rates, fractionation etc.).

(12) Defined sample

Name or specify the type of sample material to be analysed or used in other experiments. These may include but are not limited to glycoconjugates, glycosaminoglycans, N- or O-glycans, glycopeptides, glycolipids, monosaccharides, poly- and oligosaccharides.

4. LC settings

This applies for both online and offline liquid chromatography (LC) separation.

LC-MS Settings

For uploading of data to UniCarb-DB

General features — (a) Global descriptors

Date stamp	07 / 05 / 2018	*
Responsible person. Provide name,	Chunsheng Jin	*
Affiliation	Institute of Biomedicine, Gothe	*
Stable contact information	chunsheng.jin@medkem.gu.se	*

1. HPLC

HPLC manufacturer	Agilent	
HPLC brand	1100 Series	
Injector		
Injector settings		

2. Method run

Temperature (°C)	RT	
Solvent a	50 mM NH4HCO3	*
Solvent b	50 mM NH4HCO3 in 80% AcCN	*
Other solvent		
Flow rate	0.010 ml/min	*
Gradient		
Run time	67 min	
Phase	Reversed phase	*

3. Column

Manufacturer	In-house
Model	Hypercarb
Type of chromatography	Reversed phase
Type of material	porous graphitized carbon
Column diameter (mm)	0.25
Column length (mm)	100
Particle size (μm)	5

5. MS part 1

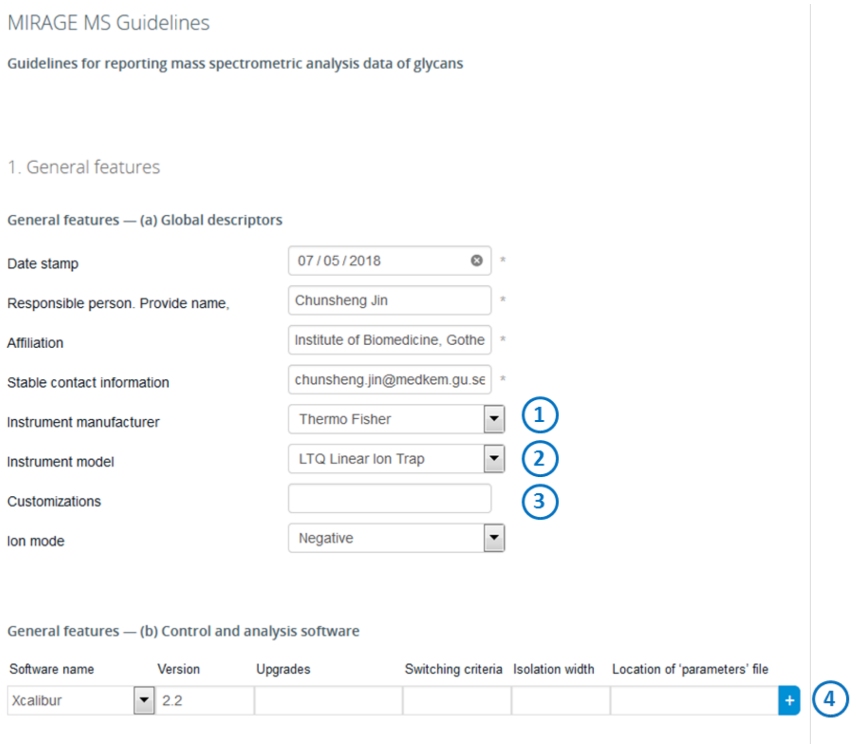

MIRAGE MS Guidelines

Guidelines for reporting mass spectrometric analysis data of glycans

1. General features

General features — (a) Global descriptors

Date stamp: 07 / 05 / 2018 *

Responsible person. Provide name,: Chunsheng Jin *

Affiliation: Institute of Biomedicine, Gothe *

Stable contact information: chunsheng.jin@medkem.gu.se *

Instrument manufacturer: Thermo Fisher (1)

Instrument model: LTQ Linear Ion Trap (2)

Customizations: (3)

Ion mode: Negative

General features — (b) Control and analysis software

Software name	Version	Upgrades	Switching criteria	Isolation width	Location of 'parameters' file
Xcalibur	2.2				

(4)

(1) The manufacturing company name for the mass spectrometer.
(2) The model name for the mass spectrometer.
(3) Any significant (i.e., affecting behavior) deviation from the manufacture's specification for the mass spectrometer.
(4) Control and analysis software

The instrument management and data analysis package name, and version; where there are several pieces of software involved, give name, version and role for each one. Also mention upgrades not reflected in the version number.

For switching criteria, it is for tandem MS only. The list of conditions that cause the switch from survey or zoom mode (MS^1) to or tandem mode (MS^n where n > 1); e.g., 'precursor ion' mass lists, neutral loss criteria and so on.

For isolation width, it refers to global or by MS level. For tandem instruments (i.e., multi-stage instruments such as triple quads and TOF-TOFs, plus ion traps and equivalents), the total width (i.e., not half for plus-or-minus) of the gate applied around a selected precursor ion m/z, provided for all levels or by MS level.

The location and name under which the mass spectrometer's parameter settings file for the run is stored, if available. Ideally this should be a URI including filename, or most preferably an LSID, where feasible. Location of file should be mentioned.

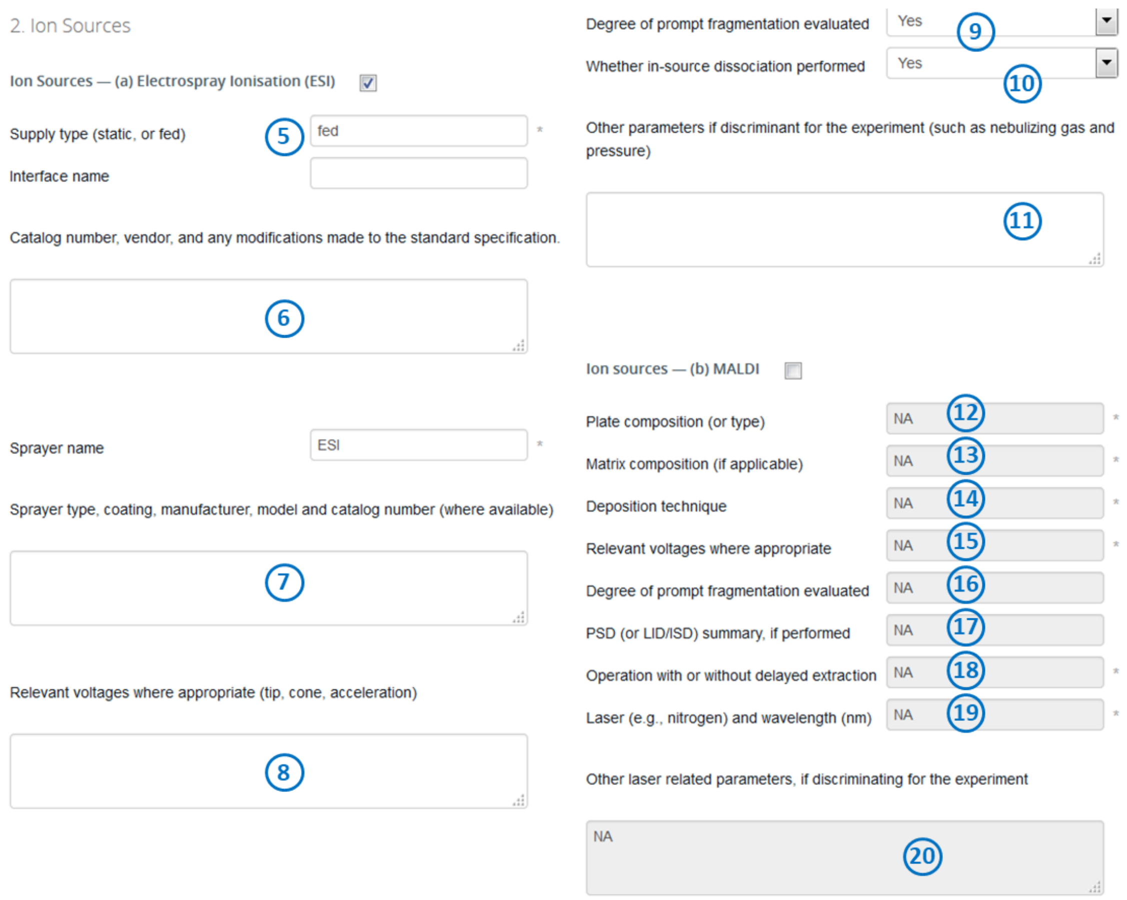

The ion sources includes (a) electrospray ionization (ESI) or (b) MALDI

For ESI,

(5) Whether the sprayer is fed (by, for example, chromatography or CE) or is loaded with sample once (before spraying).

(6) Where the interface was bought from, plus its name and catalog number; list any modifications made to the standard specification. If the interface is entirely custom-built, describe it or provide a reference if available.

(7) Where the sprayer was bought from, plus its name and catalog number; list any modifications made to the standard specification. If the sprayer is entirely custom-built, describe it briefly or provide a reference if available.

(8) Voltages that are considered as discriminating from an understood standard measurement mode, or important for the interpretation of the data. These might include the voltage applied to the sprayer tip, the voltage applied to the sampling cone, the voltage used to accelerate the ions into the rest of the mass spectrometer (mass analysis + detection) by MS level.

(9) Yes/No. If yes, provide data showing results.

(10) State whether in-source dissociation was performed (increased voltage between sample orifice and first skimmer).

(11) Where appropriate, and if considered as discriminating elements of the source parameters, describe these values.

For MALDI,

(12) The material of which the target plate is made (usually stainless steel, or coated glass); if the plate has a special construction.
(13) The material in which the sample is embedded on the target (e.g., 2,5-dihydroxybenzoic acid (DHB)).
(14) The method of laying down (matrix and) sample on the target plate (including matrix concentration and solvents applied); for example, matrix+sample in single deposition; or matrix, then matrix+sample (if several matrix substances are used, name each), Recrystallization using volatile solvent; where chromatographic eluent is directly applied to the plate by apparatus, or for other approaches, describe the process and instrumentation involved very briefly and cross-reference.
(15) Voltages considered as relevant for the interpretation of the data. This might include the grid voltage (applied to the grid that sits just in front of the target), the acceleration voltage (used to accelerate the ions into the analyzer part of the mass spectrometer (mass analysis + detection), etc.
(16) Yes/No. If yes, provide data showing results.
(17) Confirm whether post-source decay, laser-induced decomposition, or in-source dissociation was performed; if so provide a brief description of the process (for example, summarize the stepwise reduction of reflector voltage).
(18) State whether a delay between laser shot and ion acceleration is employed.
(19) The type of laser and the wavelength of the generated pulse (in nanometers).
(20) Other details of the laser used to irradiate the matrix-embedded sample if considered as important for the interpretation of data; this might include the pulse energy in microJoules, focus diameter in microns, attenuation details, pulse duration in nanoseconds at full-width half maximum, frequency of shots in Hertz and average number of shots fired to generate each combined mass spectrum.

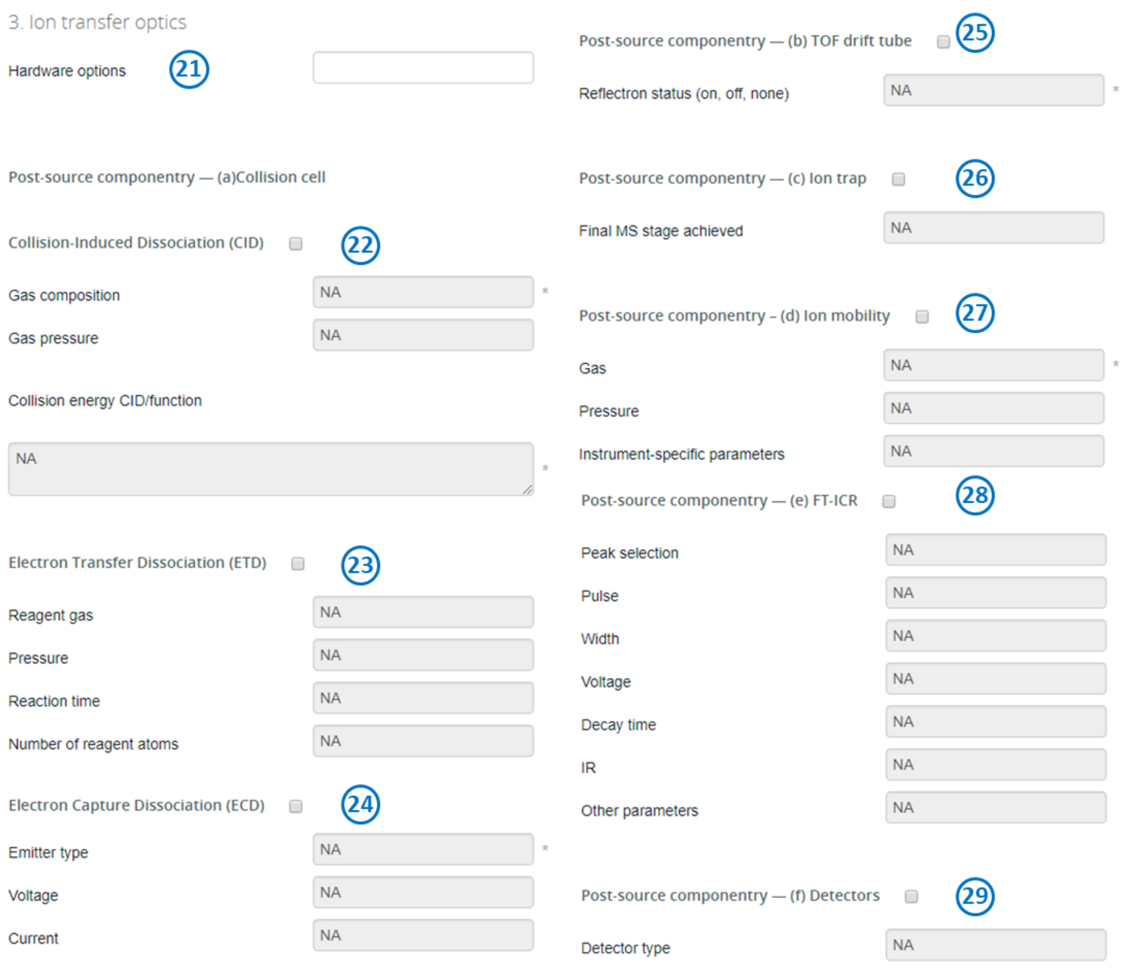

3. Ion transfer optics

Hardware options (21)

Post-source componentry — (a)Collision cell

Collision-Induced Dissociation (CID) (22)

Gas composition: NA *

Gas pressure: NA

Collision energy CID/function

NA *

Electron Transfer Dissociation (ETD) (23)

Reagent gas: NA

Pressure: NA

Reaction time: NA

Number of reagent atoms: NA

Electron Capture Dissociation (ECD) (24)

Emitter type: NA *

Voltage: NA

Current: NA

Post-source componentry — (b) TOF drift tube (25)

Reflectron status (on, off, none): NA *

Post-source componentry — (c) Ion trap (26)

Final MS stage achieved: NA

Post-source componentry – (d) Ion mobility (27)

Gas: NA *

Pressure: NA

Instrument-specific parameters: NA

Post-source componentry — (e) FT-ICR (28)

Peak selection: NA

Pulse: NA

Width: NA

Voltage: NA

Decay time: NA

IR: NA

Other parameters: NA

Post-source componentry — (f) Detectors (29)

Detector type: NA

(21) Hardware options refer to e.g. “simple” quadrupoles, hexapoles, stacked ring electrodes, TOF, …

(22) For collision-induced dissociation (CID)

The composition and pressure of the gas used to fragment ions in the collision cell (TOF-TOF, linear trap, Paul trap, or FT- ICR cell) should be indicated.

Collision energy CID/function refers to the specifics for the process of imparting a particular impetus to ions with a given m/z value, as they travel into the collision cell for fragmentation. This could be a global figure (e.g., for tandem TOFs), or a complex function; for example a gradient (stepped or continuous) of m/z values (for quads) or activation frequencies (for traps) with associated collision energies (given in eV).

(23) For electron transfer dissociation (ETD)

Reagent gas, pressure, reaction time, and number of reagent ions should be filled in.

(24) Electron capture dissociation (ECD)

Emitter type, voltage, and current should be filled in.

(25) TOF drift tube

Whether a Reflectron is present, and if so, whether it is used. Depending on the type of instrument provide exact details on the reflectron mode (e.g. V or W mode).

(26) Ion trap

The final MS level achieved in generating this data set with an ion trap or equivalent (e.g., MS^10).

(27) Ion mobility

The gas, pressure, and instrument-specific parameters (e.g. wave velocity/height depending on the particular vendor's options for tuning this component) should be filled in.

(28) FT-ICR

Peak selection, pulse width, voltage, decay time, IR and other important experiment parameters should be filled in.

(29) Detectors

Need to define detector type if non OEM detector were used (e.g. microchannel plate, channeltron etc.).

6. MS part 2

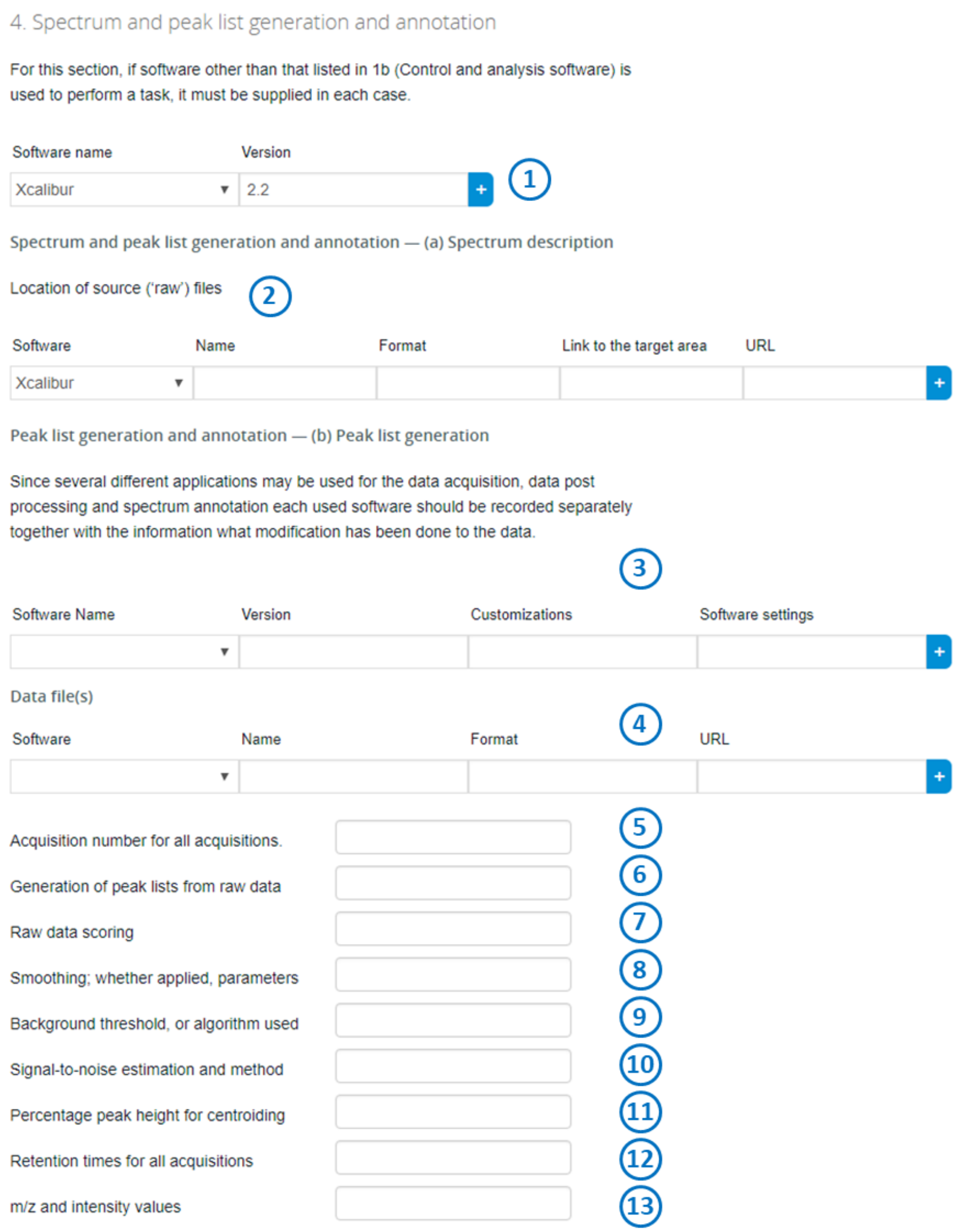

4. Spectrum and peak list generation and annotation

For this section, if software other than that listed in 1b (Control and analysis software) is used to perform a task, it must be supplied in each case.

Software name	Version	
Xcalibur	2.2	(1)

Spectrum and peak list generation and annotation — (a) Spectrum description

Location of source ('raw') files (2)

Software	Name	Format	Link to the target area	URL
Xcalibur				

Peak list generation and annotation — (b) Peak list generation

Since several different applications may be used for the data acquisition, data post processing and spectrum annotation each used software should be recorded separately together with the information what modification has been done to the data.

(3)

Software Name	Version	Customizations	Software settings

Data file(s)

(4)

Software	Name	Format	URL

Acquisition number for all acquisitions.		(5)
Generation of peak lists from raw data		(6)
Raw data scoring		(7)
Smoothing; whether applied, parameters		(8)
Background threshold, or algorithm used		(9)
Signal-to-noise estimation and method		(10)
Percentage peak height for centroiding		(11)
Retention times for all acquisitions		(12)
m/z and intensity values		(13)

(1) For this section, if software other than that list in **Control and analysis software** is used to perform a task, the producer, name and version of that software must be supplied in each case.

(2) The location and filename under which the original raw data file from the mass spectrometer is stored, if available.

Give the type of the file where appropriate, or else a description of the software or reference resource used to generate it. Due to the nature of the raw files (proprietary formats, no open source software, licensing, etc), the validation of raw data can only be possible if the information is provided in an open XML format (mzXML, mzData, mzML). Input either a spot number or some other form of coordinates if more appropriate, that link the spectrum to the

analyzed area of the sample (2D imaging). Ideally this should be a URL or filename, or most preferably an LSID, where feasible.

(3) For peak list generating software,

This includes the name of the software, the version number, any changes made to the original program code that may affect the results and any settings made in the software that may affect the results (e.g. thresholds).

(4) Provide information about the produced data file. This includes the name of the software, the name of each file, the file format, the availability of the file and if applicable the URL to access the file.
(5) Where available, the reference numbers of all the scans (as numbered in the raw file) that were combined to produce a peak list, the total number of acquisitions combined to produce the peak list, and whether the peak list was produced by summing or averaging the scans that are listed.
(6) The total ion count or S/N threshold for a spectrum and the minimum number of ions detected in that scan, for it to be a candidate for grouping in a peak list; plus the mass tolerance (Da) on the precursor ion masses for MS/MS spectra.
(7) Describe method and software for selection of peaks for inclusion in the peaklist.
(8) Any peak smoothing should be described, along with the parameters supplied to the algorithm.
(9) The ion abundance or S/N cut-off used to filter background noise; or a description of the algorithm used to gate the noise, if complex.
(10) The ratio of signal to noise for each significant peak in a peak list; significance is defined as being above a given ion abundance(which should be supplied) or being otherwise of interest; the method of calculation should also be named (if available).
(11) The percentage peak height at which centroids are calculated; if a more complex algorithm is used to perform the process, it should be named here.
(12) The times relative to the start of the MS run for all acquisitions that were combined in the peak list so that those acquisitions may later be correlated to a chromatogram (continuously-fed electrospray sources only).
(13) The actual data (m/z versus ion abundance); as described in the preceding sections.

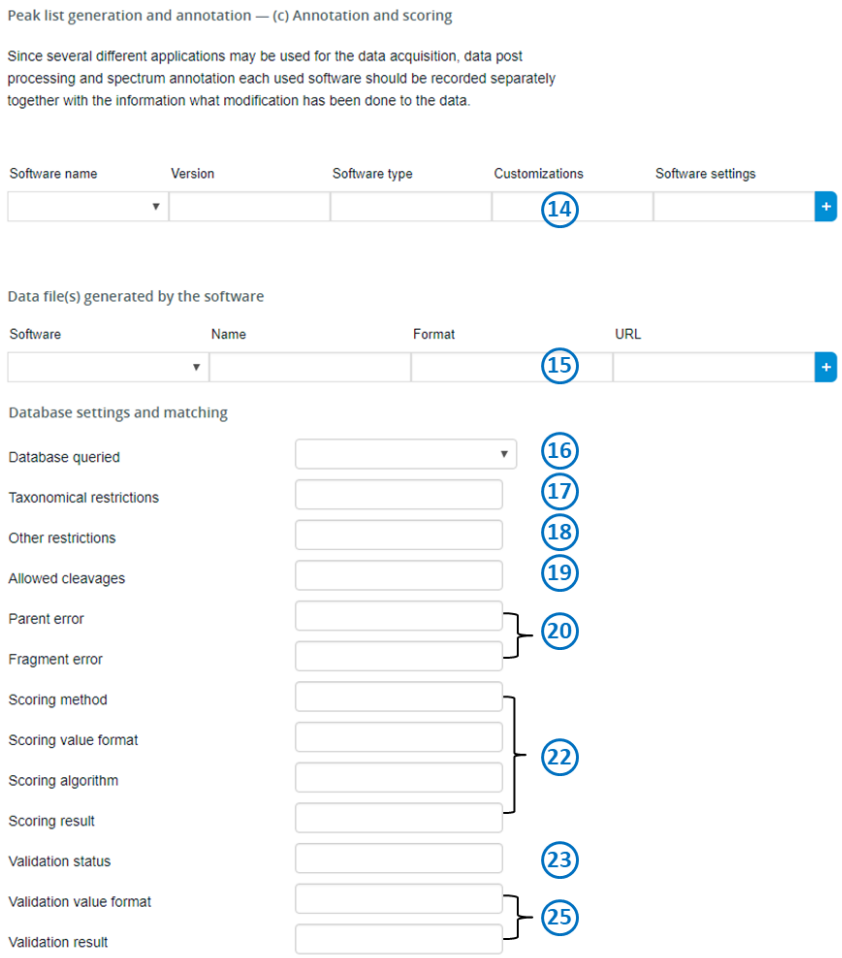

(14) This includes the name of the software, the version number and type of data processing that was performed with the software. Any changes made to the original program code that may affect the results. Any settings made in the software that may affect the results (e.g. thresholds).

(15) Information about the annotation data file. This includes the file format, the availability of the file and if applicable the URI to access the file.

(16) List of databases used for the annotation of the data. Also specify databases version, annotation date and number of entries.

(17) List of species the search was limited to.

(18) Other settings to the software that filtered out certain sequences from the database (e.g. allow only certain glycan types (N-Glycan) or restriction by composition). This also includes the usage of threshold for scoring values.

(19) List of allowed cleavages for the annotation run (A, B, C, X, Y, and Z). This includes also the number of allowed cross-ring cleavages and glycosidic cleavages.

(20) Mass accuracy settings of both precursor and fragment for the annotation run.

(21) Used scoring function with references to the algorithm and of software.

(22) Validation status for all glycan structures, specify if accepted without post-processing of database/de-novo interpretation or if manually accepted or rejected.

(23) Confirmation of preliminary assignment with tandem MS data. In the case of glycan tandem/multistage MS profiling (MSn) describe the number of fragmentation stages and m/z values associated to the identified glycan.

Glycoworkbench file

This protocol is used to deposit annotated glycan structures, peak list, and other related mass spectral information (e.g., annotation) into an integrated file (glycoworkbench workspace file, .gwp format). The GWP file contains all content that needed to present in Unicarb-DR (http://unicarb-dr.biomedicine.gu.se/).

1. Download and install the software (https://code.google.com/archive/p/glycoworkbench//).
2. Download the sample Glycoworkbench file from Unicarb-DR (http://unicarb-dr.biomedicine.gu.se/generate).
3. Open downloaded sample Glycoworkbench file and draw the first structure in Glycoworkbench.

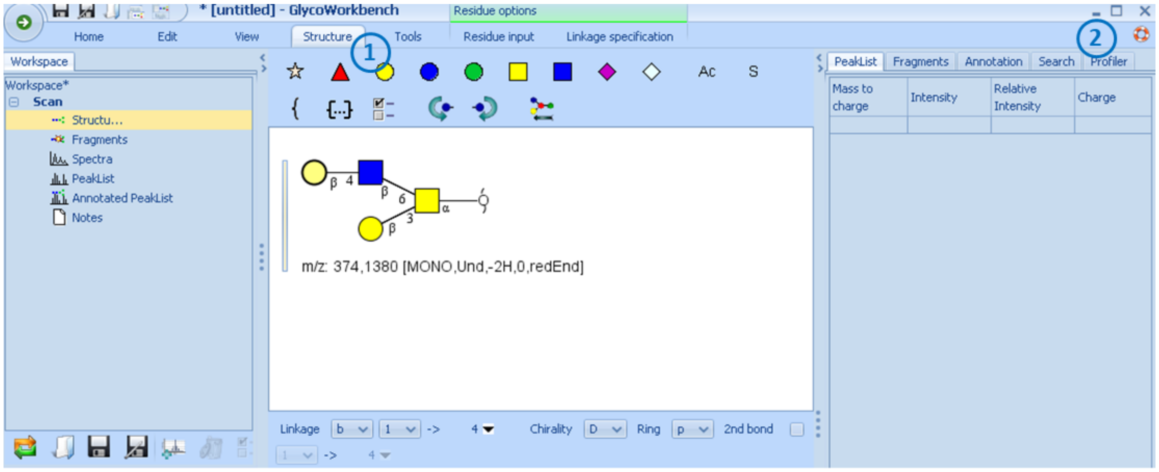

(1) Using Structure function to draw the structures. For uncertain residue(s), just put e.g. hexose (blank circle). For uncertain linkage, put question mark.

(2) The detailed manual can found here.

4. Set the calculated mass close to observed mass

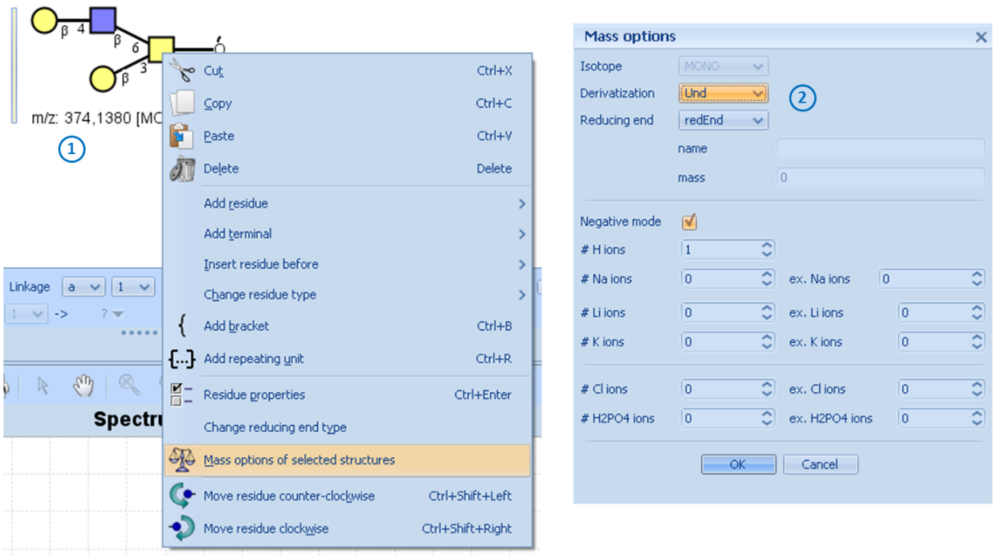

(1) Select the structure and right click. Choose **Mass options of selected structures**.

(2) Choose the settings for observed mass. Click **OK**. In case of positive mode such as [M-H+2Na]$^{+}$, it can be expressed as one H ion with two ex Na ions. For the negative ions, [M-2H+Na]-, it can be expressed as two H ion with one ex Na ion.

5. Change the scan name to observed mass.

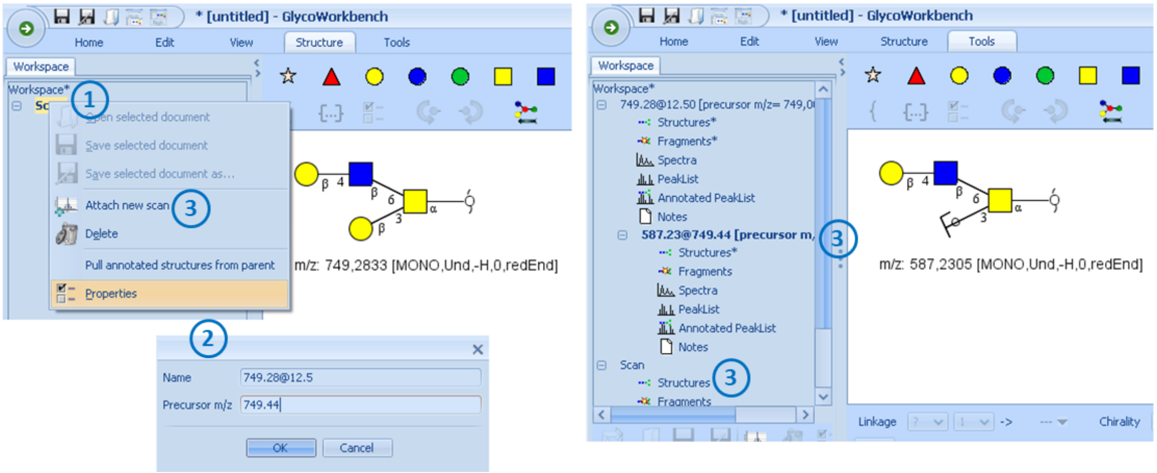

(1) Right click **Scan** and choose **Properties**.

(2) In the column **Name**, add calculated mass with either retention time or G.U. value separating with @ (e.g., 749.28@12.50 or 749.28@gu5.2). In the column **Precursor m/z**, add observed mass. Click **OK**. **NOTE**: use period as decimal point rather than comma.

(3) If MS3 is available, a new scan should be added by choosing **Attach new scan**. Then fragmental structure can be drawn in the new scan window.

If one wants to add second structure, right click **Workspace** and click **Attach new scan**.

6. Input peak list.

The MS/MS peak list is a two-column matrix containing peaks (m/z values) and ion abundance of each peak. The peak list refers to any kind of centroid data, which are not in profile mode, i.e. do not have continuous m/z data. Preferably, the list should contain 100-200 mass peaks.

For Thermo raw data, open raw file in **Qual Browser** which contains the target MS/MS spectra. In Spectrum window, right click and choose **View**>**Spectrum List.** Right click and choose **Display Options**. Select **Centroid** and input 100 or tick "All peaks". Click OK. To export peak list in Spectrum window, right click and choose **Export**>**Clipboard (Exact Mass)**.

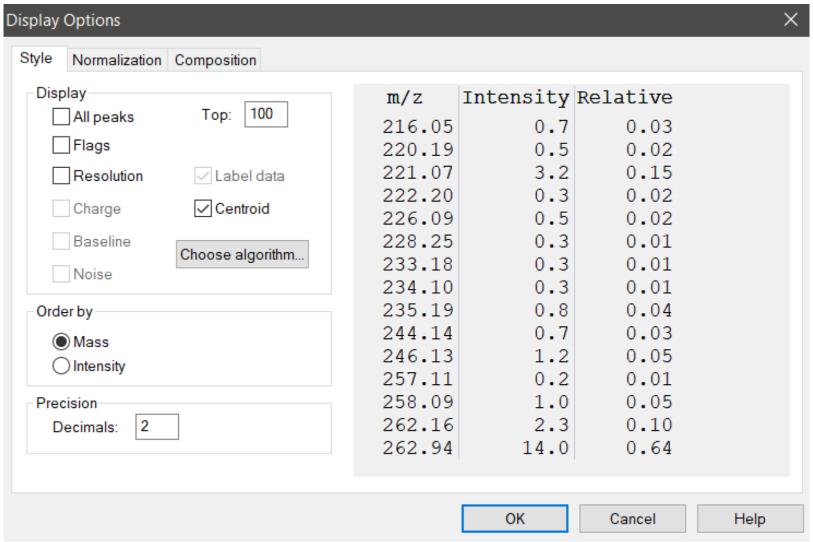

For Waters raw data, open raw file in MassLynx and display MS/MS spectra in spectrum window. In the spectrum window, go to **Process**>**Center…**. In the **Min peak width at half height (channels)**, input 5 or higher values so that the peak list would contain 100-200 top peaks. In order to export peak list, click **Edit** and choose **Copy Spectrum List**.

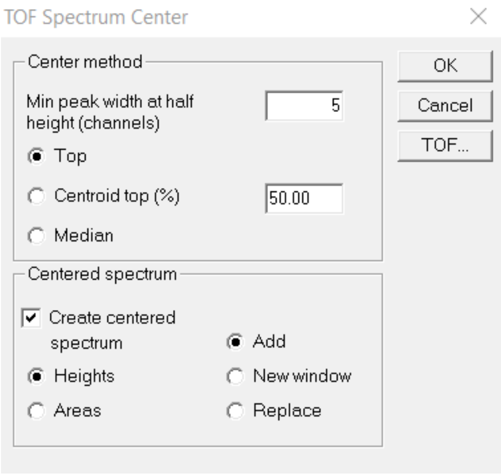

To input the peak list to Glycoworkbench,

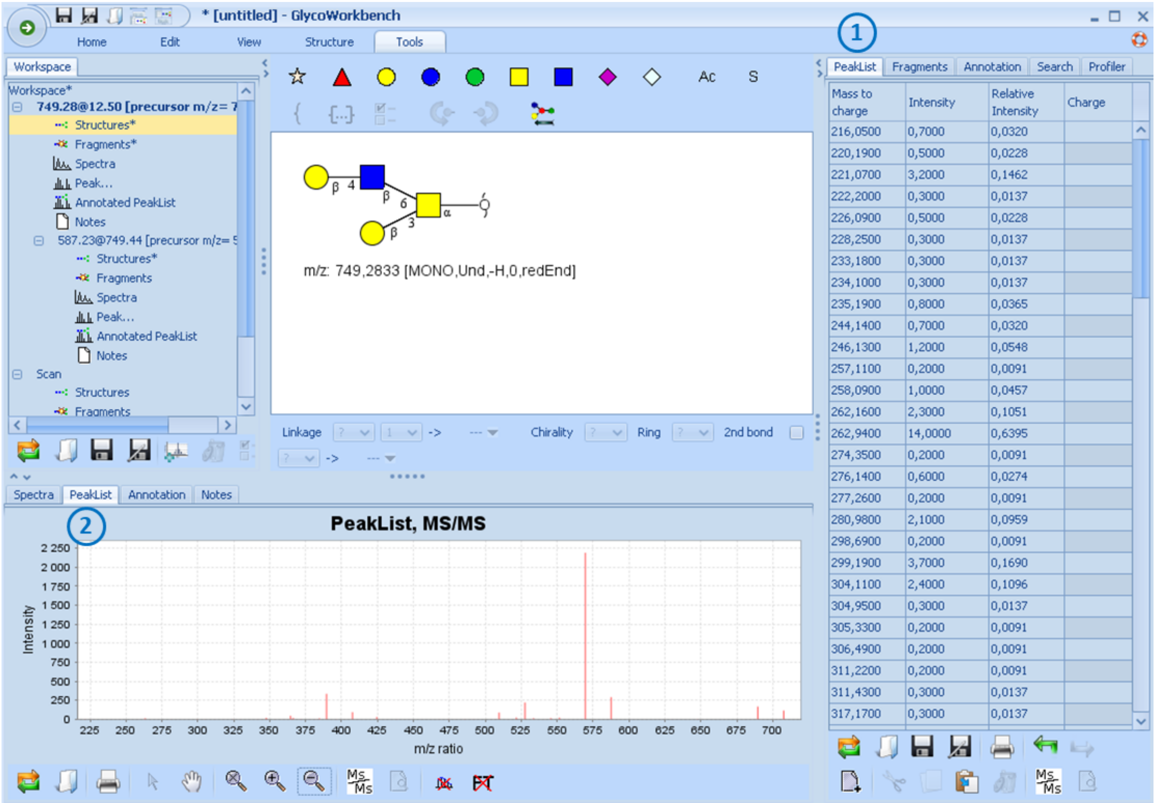

(1) Click **PeakList** and select the first cell under **Mass to charge**. Right click and select **Paste**.
(2) Click **PeakList**, the corresponding MS/MS spectrum will appear in Spectrum window.

7. Annotation

To annotate the peaks, select the structure and go to **Tools**. Select **Annotation**>**Annotate peaks with fragments from selected structures**. The **Fragment options** window appears.

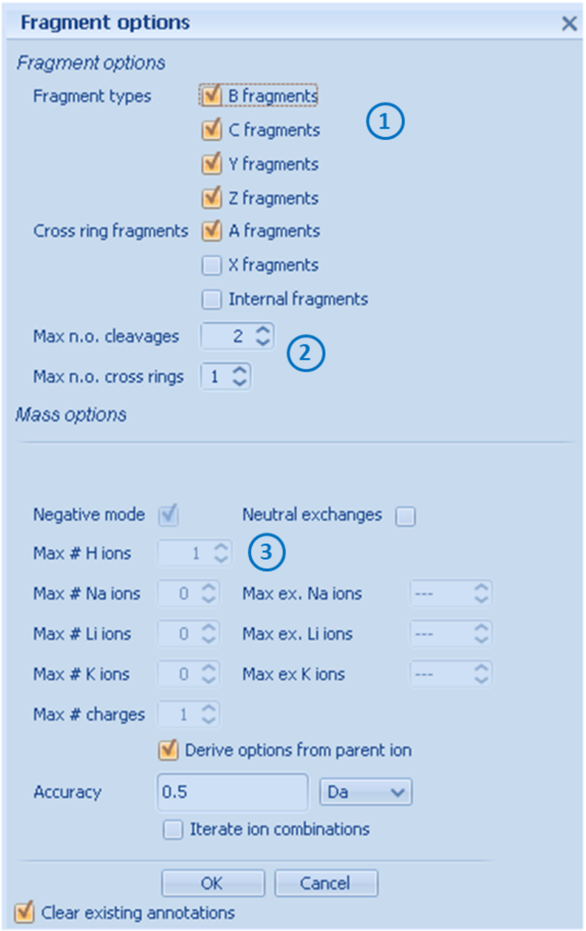

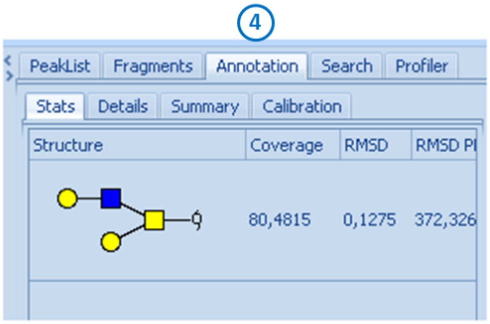

(1) For MS/MS spectra obtained from positive-ion mode, no cross ring fragments should be selected in general. For MS/MS spectra obtained from negative-ion mode, only A fragments should be selected for non-sialylated oligosaccharides; both A and X fragments should be selected for sialylated oligosaccharides.

(2) Depending on molecular fragmentation technology, specify the maximal number of cleavages. For CID, it allows two maximal glycosidic cleavages and one cross-ring cleave.

(3) If the precursor ions are doubly charged (e.g., $[M-2H]^{2-}$) when drawing the structure, both singly and doubly charged fragment ions will be annotated. If the precursor ions are singly charged (e.g., $[M-H]^{-}$), only singly charged fragment ions will be assigned.

(4) Annotation results will appear in right panel of Glycoworkbench after click **OK** in **Fragment options**.

8. Validation of annotation

The result of annotation needs validation before uploading to UniCarb-DR to remove ambiguous assignments.

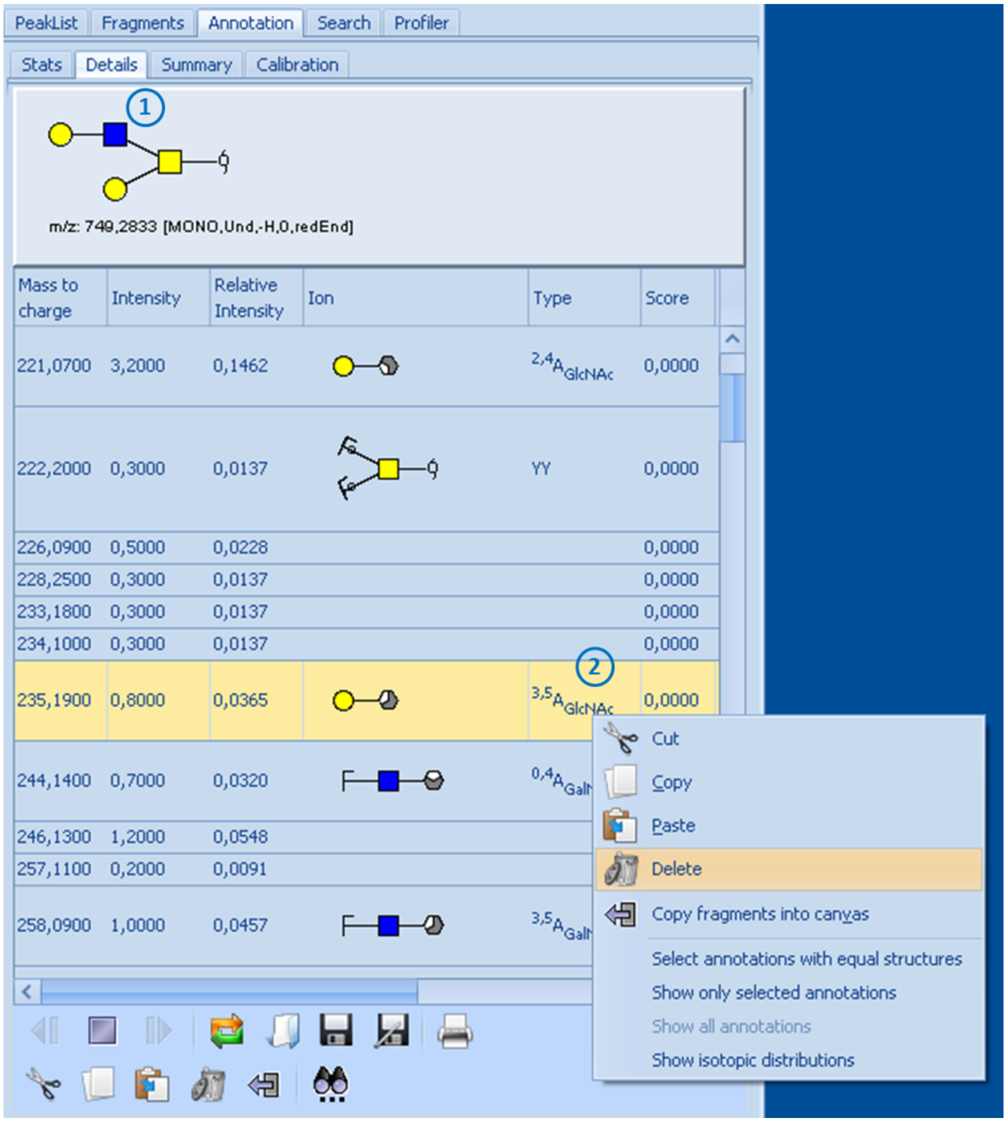

(1) Select **Annotation**>**Details**, where detailed annotation can be found.

(2) To remove ambiguous annotation (mainly cross-ring fragments), click the structure that will be removed and right click. Select **Delete**, which only remove type of fragment (e.g., $^{3,5}A_{GlcNAc}$) rather than fragment ions from list. Usually, $^{0,2}A$, $^{0,4}A$ and $^{2,4}A$ cleavages are kept. For N-glycans, $^{0,3}A$ of βMan and $^{1,3}A$ of αMan residues are also kept. For sialylated structure, $^{0,2}X$sialic acid ions are considered if present.

9. Note of annotated structure

In sample Glycoworkbench file downed from Unicarb-DR (http://unicarb-dr.biomedicine.gu.se/generate), there is **Note** section to record all information of selected structure. The content of Note section can be copied and pasted.

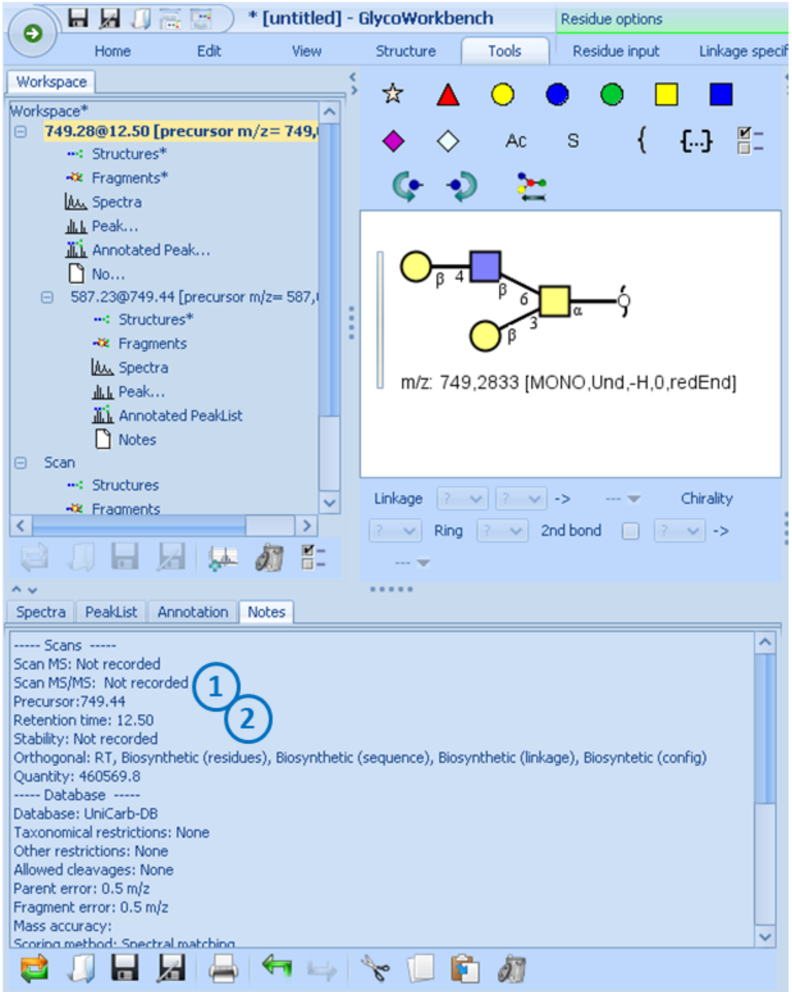

(1) At least, the m/z value of precursor ions should be recorded.
(2) At least, the retention time or g.u. values should be recorded.

10. Save the file as Glycoworkbench workspace file (.gwp) if no more structure is added.

Uploading to Unicarb-DR

This step is to upload MIRAGE File and Glycoworkbench files (.gwp).

1. Sing up or log in Unicarb-DR.
2. Click the user name>**Submit data**.

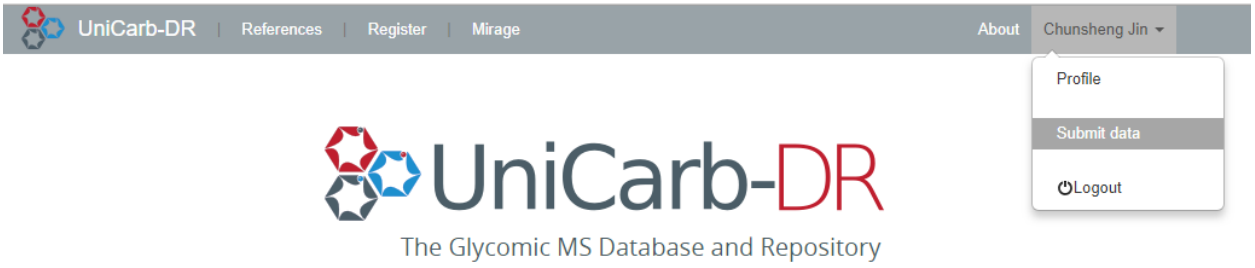

3. Upload MIRAGE file and Glycoworkbench file.

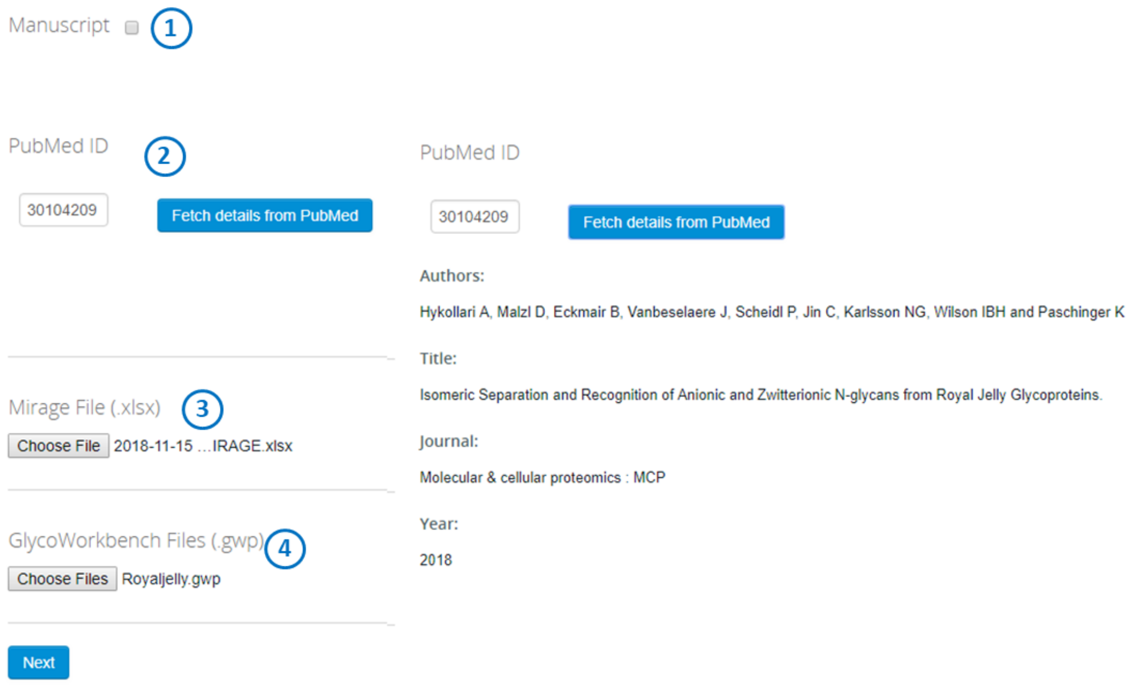

(1) Tick it if it is a manuscript.
(2) Put PMID if the manuscript was published and click **Fetch details from PumMed**. The brief description will appear.
(3) Upload MIRAGE file.
(4) Upload Glycoworkbench file. Click **Next**.

4. A window for the user's inspection will appear. If no mistake is detected, click **Submit**.

SUPPLEMENTARY METHODS

System overview and implementation

UniCarb-DR repository is based on the UniCarb-DB database format[1, 2], adopted to include tables and layouts for MIRAGE information. The repository design is based on a PostgreSQL as database manager system. The UniCarb-DR web application is supported by the Play Framework (https://www.playframework.com/). The Play Framework makes use of the MVC paradigm, where the elements of an application adopt one of three roles: Model, View or Controller. The Model is written in Java and represents the data and how the data is manipulated. The View is the layer that is displayed to users in the web interface. In UniCarb-DR, the View is written in Scala, JavaScript and implements the Jquery, Bootstrap and SpeckTackle libraries for data visualization. The Controller layer, also written in Java, controls the data that flows to the model and updates the View when the data change in response to user actions.

Testing of the MIRAGE glycomic workflow

In this review, we propose a workflow to collect, process and store experimental data in compliance with the MIRAGE MS and sample preparation guidelines a UniCarb-DR (DR = Data Repository) that benefits from the previous developed UniCarb-DB framework of quality LC-MS/MS data and structural assignments[1, 2]. UniCarb-DR incorporates both the MIRAGE MS and sample preparations guidelines. It also provides an electronic submission tool, guiding users for initial data validation to ensure all required information is provided. Data is entered in a structured form (template, http://unicarb-dr.biomedicine.gu.se/generate) that can be submitted to UniCarb-DR together with GlycoWorkbench files, including structures, spectra, fragmentation annotation and meta-data with scoring parameters, spectral quality and the use of orthogonal methods for structural assignments.

In order to develop and test the MIRAGE parameter on-line form and the submission tool, we selected beta-test sites that generated glycomic LC-MS^2 and MS^2 from *N*-linked, *O*-linked and proteoglycan type protein oligosaccharides ((http://unicarb-dr.biomedicine.gu.se/references). MIRAGE data spreadsheets were generated via the described on-line submission form available at http://unicarb-dr.biomedicine.gu.se/generate, where LC parameters also were recorded. Generated spreadsheets from this submission are available in supplementary material. Individual centroided MS^2 spectra were copied manually into GlycoWorkbench[3] .gwp files together with the identified structures assigned from peak matching or manual interpretation Examples of Glycoworkbench files is also available in supplementary material. Structures were assigned based on MS^2 spectra and/or retention time and the quality of matching was manually validated.

Global MIRAGE specific controlled vocabulary

In the web form, the user can select predefined glycospecific MIRAGE information. In practise, it mostly relates to specific pretreatment of samples (exoglycosidases, permethylation etc) included in the MIRAGE sample preparation guidelines or in the MS section. A few resources cover this

information such as GlycoSuiteDB[4] that is no longer available but now included in GlyConnect (https://glyconnect.expasy.org/) and GlycoDigest (https://glycoproteome.expasy.org/glycodigest/). The treatment list is available in supplementary Spreadsheet. Being aware that current information about treatments in glycomics is evolving, UniCarb-DR will also accept user-defined treatments as submitted in the spreadsheet. This will expand the controlled vocabulary of specific treatments in glycomics as submission to UniCarb-DR progresses. At some stage, settling on a more rigorous maintenance of the treatment-controlled vocabulary may become necessary.

Recording of MIRAGE MS^n specific metadata

The MIRAGE guidelines require that MS information for individual structures should be recorded for each structure. By implementing Glycoworkbench as part of a UniCarb-DR submission, the .gwp file format can be used in compliance with MIRAGE. In addition to structural recording and the inclusion of fragment lists with *m/z* (preferentially converted to centroid data) and ion abundances, Glycoworkbench automatically calculates theoretical masses based on a user-defined charge state, ion mode and derivatization. Glycoworkbench also has modules to calculate and match theoretical fragments with observed ones with a basic score. However, MIRAGE parameters such as "observed precursor ion *m/z*", "orthogonal methods" that have been used for identifying individual structures, "scoring" and "validation methods" of fragment data are not recorded in the .gwp file. We propose a model where this information can be included in the 'Notes' section in the Glycoworkbench file (Figure 3).

Orthogonal methods

In addition to MS, orthogonal methods are classically used in order to fully characterize a glycan structure. To account for this information we propose that the sample preparation methods defined above (supplementary material) also serve as the controlled vocabulary for orthogonal validation of individual structures. Of course this list also needs to be expanded by input from the community and associated with other glycomic experimental data.

Since the assignment of structures is often based on previous knowledge about the samples, we propose to expand the orthogonal method list with four additional items; this is to capture various aspects of information not necessarily obtained by MS. These are:

1) Residues: Type of monosaccharide that constitutes the structure. MS is usually not sufficient for distinguishing between constituting isomeric monosaccharide units in a structure. A typical question is to establish if previous or biosynthetic knowledge was used in order to assign the monosaccharide composition. If for example, a Mannose is assigned to a certain position rather than the more generic Hexose, is it because of prior knowledge about the sample? This orthogonal method is captured as *Biosynthetic(residue).*
2) Primary Sequence: If the order of monosaccharide units in the structure is assumed based on previous or biosynthetic knowledge, i.e. if the primary sequence of an *N*-linked

oligosaccharide core is put down as Hex-(Hex-)Hex-HexNAc-HexNAc, without evidence from MS, the use of this non MS generated additional information should be captured as *Biosynthetic(sequence).*

3) Linkage position: The linkage position in an assigned structure. For example, is Fuc assigned as Fuc**1-2**Gal based on prior or biosynthetic knowledge of blood group H that was shown to be present in the samples? This orthogonal method is captured as *Biosynthetic(linkage).*
4) Linkage configuration: The linkage configuration (usually α and β) in an assigned structure. For example, is Fuc assigned as Fuc**α**1-2Gal based on prior or biosynthetic knowledge of blood group H that was shown to be present in the sample? This orthogonal external information for assigning structures should be recorded as *Biosynthetic(config).*

If only MS is used to assign oligosaccharide structures, we believe that the default should be to include these 4 methods in the MIRAGE file. This is to acknowledge that MS is often not enough for a total characterization of a carbohydrate structure.

Scoring of MSn fragmentation data

The first MIRAGE guideline for MS was published in 2013 (23378518) and was based on state of the art glycomic analysis. At the time there were few e-tools used for the interpretation of MS data and scoring of the fragment spectra. Hence, the guidelines only requested the recording of the number of unmatched peaks for each spectrum. This information can be obtained using the peak-matching tool of Glycoworkbench, and could be captured for MIRAGE compliance from this file. However, since the publication of the guidelines, more sophisticated methods for measuring the quality of fragment ions have been developed. We propose to expand on the current guidelines to include this qualitative information. Rather than relying on the number for unmatched peaks, we record the actual scoring. For this we request that the report should include a defined vocabulary for the different types of scoring used in glycomics. Based on our experience in scoring spectra for structural assignment the following 4 items should be included in a MIRAGE report:

1) Scoring method: Answers the question: which method was used? Options would include *manual* interpretation or software aided interpretation such as *de-novo sequencing* methods, *spectral matching or matched/unmatched peaks.* For the scoring method to be relevant there is also a potential need to include:
    i. Errors of the mass allowed for precursor ion and fragments.
    ii. If (and which) database has been used for the scoring
    iii. Restrictions i.e. in type of fragments searched, species exclusion or other exclusion from the database
2) Scoring algorithm: Answers the question: Is there a particular algorithm used to perform the scoring? For example, the *normalized dot product* is the most common algorithm for spectral matching.
3) Scoring result: Answers the question: what is the value (or values) output by the scoring

method?

4) Scoring value format: The experience from proteomics is that a scoring result may not be a single value, so we propose that the format of the result is a string on values (text separated by comma), and that the scoring value format is a controlled vocabulary that defines the layout of the scoring result.

We have for several years defined and used internally a scoring named *UniCarb-DB triplet*. This score is based on the value of the normalized dot product and increased (i) if the matched structure is *identical* to the proposed structure, (ii) if it shares the same *sequence* or if it shares the same *composition.* Information about the rank of the proposed structure in the search result list is also considered. We introduce the triplet notation with an example: "0.99,identical,1" where *0.99* is the dot product score, *identical* indicates 100% similarity between the matched and proposed structures, and *1* indicates the rank of the right answer in the search result list. Other values for the first item can be *no-match*. The scoring value format of *UniCarb-DB triplets* should be defined in the controlled vocabulary for scoring.

Validation of structures

The objective of the validation is to give an overview of the structural features that could be determined by MS vs. other information. MS fragmentation is expected to provide primary sequence information. However, we need to use orthogonal methods to determine a full structure and connect it with biological function. The MIRAGE guidelines require information on how a structure was validated. However, the means for how to do so are not defined. Options should cover *manually* or *automatically,* but also *other (*eg false discovery rate). Furthermore, information about the MS^n level used for validation and their corresponding results are informative. The validation result format should be similar to that of the scoring, i.e. recorded as a string of values separated by commas.

Several features of a structure need to be validated including monosaccharide composition (C), primary sequence (S), linkage position (L), and linkage configuration (C). We suggest the definition of a format notation, and to set the default as the *manual CSLC*-format to capture how conclusive the MS and fragment data are for the structure that is proposed. If it is found that the fragment data fully supports each of these 4 items (composition, sequence, linkage and configuration) for a fully assigned structure containing monosaccharide speciation, linkages and configuration, the validation results should be 1,1,1,1. If it is found that nothing is substantiated the results instead should be 0,0,0,0. For easy manual evaluation we propose the following reasoning with a hexasaccharide as an example:

1) Monosaccharide composition (C): The mass of an oligosaccharide provides information about the composition, but is the MS itself conclusive to identify isomeric monosaccharide units? With a manual validation it is always a matter for the researcher to judge, but we can try to provide some guidelines based on our own experience. For a hexasaccharide

consisting only of 3 Hexoses and 3 *N*-acetylhexosamines, it is unlikely that only MS and MS^2 data will provide information about the type of Hex or HexNAc isomer. Hence the first C value in the validation results should be “0” if the proposed structure suggests specific monosaccharide units for Hex and HexNAc (like Man and GlcNAc). Another example is a hexasaccharide with a composition of $Hex_2HexNAc_2Fuc_1NeuAc_1$. If this is structure was found in previously referenced source, where both fucose and N-acetylneuraminic acid are known to be present, and fragmentation data provides clear evidence that masses corresponding to Fuc and NeuAc residues, one could argue that presence of 2 of the 6 monosaccharides has been validated, because of the lack of isomeric residues in the source. Hence, the validation result should be 2/6 = 0.33 if the proposed structure also contains speciation of Hex (e g Man and/or Gal) and HexNAc (e g GlcNAc) units.

2) Primary sequence (S): How well does the fragmentation data support the proposed sequence? For a hexasaccharide there are 5 linkages that need to be identified. A quick way to validate this is to check if there is any evidence for all glycosidic fragments in the spectra (validation result =1). If one fragment is lacking but still recorded (‘guessed’) in the proposed structure, the primary sequence (S) validation value should be 4/5 = 0.8. In order to perform this manually, we propose the use both single and internal glycosidic fragment assignments. Note that only because all glycosidic linkages are detected, the sequence may not be conclusive and other sequences may also fit the spectra.
3) Linkage position (L): Is there evidence for a specific fragmentation of linkage position? In a hexasaccharide, there are 5 linkage positions that should be determined (assuming the permanence of a link via the anomeric C-1 carbon). If all of the linkages are assigned in the proposed structure but linkage specific fragmentation evidence (usually cross ring fragmentation) is lacking for one of them, the linkage position validation should be 4/5 = 0.8. Note that assignment of cross-ring fragments may be unequivocal.
4) Linkage configuration (C): Usually MS is not the ultimate method to determine α or β configuration, so if these are recorded in all the linkages for a proposed structure the linkage configuration default validation result should be “0”. One could argue that MS may contain this information if for instance the fragmentation (fragment ions and/or ion abundance) is found to be different for an α or β isomer. This could be the case for instance using MS^n methodology[5] or configuration specific fragmentation using ion mobility[6].

It should be pointed out that using this format, orthodox reporting of structures from fragment data provided in the form of numbers of Hex and HexNAc and primary sequence data (all glycosidic fragments) with unknown linkage positions and configurations, are validated with a score of 1,1,1,1. The same structure, recorded instead with Man, Gal, GlcNAc and GalNAc residues and fragments covering all glycosidic linkages, but recorded with linkage position and configuration without MS evidence, will have a validation score of 0,1,0,0. Hence, the validation is not only capturing the quality of the MS data, but also how orthogonal was utilized for interpretation. Other ways of validation of structures for glycomic analysis will inevitably be developed. We assume

that our implemented system for MIRAGE recording is flexible enough to incorporate these.

SUPPLEMENTARY REFERENCES

1. Campbell MP*, et al.* Validation of the curation pipeline of UniCarb-DB: building a global glycan reference MS/MS repository. *Biochim Biophys Acta* **1844**, 108-116 (2014).

2. Hayes CA*, et al.* UniCarb-DB: a database resource for glycomic discovery. *Bioinformatics* **27**, 1343-1344 (2011).

3. Damerell D, Ceroni A, Maass K, Ranzinger R, Dell A, Haslam SM. The GlycanBuilder and GlycoWorkbench glycoinformatics tools: updates and new developments. *Biol Chem* **393**, 1357-1362 (2012).

4. Cooper CA, Harrison MJ, Wilkins MR, Packer NH. GlycoSuiteDB: a new curated relational database of glycoprotein glycan structures and their biological sources. *Nucleic Acids Res* **29**, 332-335 (2001).

5. Ashline DJ, Zhang H, Reinhold VN. Isomeric complexity of glycosylation documented by MSn. *Anal Bioanal Chem* **409**, 439-451 (2017).

6. Hofmann J, Hahm HS, Seeberger PH, Pagel K. Identification of carbohydrate anomers using ion mobility-mass spectrometry. *Nature* **526**, 241-244 (2015).
