# Supplementary material for: Towards a standardized bioinformatics infrastructure for N- and O-glycomics
Source: Nat Commun. 2019 Jul 22;10:3275. doi: 10.1038/s41467-019-11131-x (PMC6796180; doi:10.1038/s41467-019-11131-x)
Supplement: Supplementary file 2 — Description of Additional Supplementary Files [file 41467_2019_11131_MOESM2_ESM.docx]

**Description of Supplementary Files**

**File Name:** **Supplementary Data 1**

**Description:** Example of a prefilled MIRAGE spreadsheet generated from the webform available at <https://unicarb-dr.biomedicine.gu.se/generate>.

**File Name:** **Supplementary Data 2**

**Description:** Glycomic specific treatments and orthogonal methods vocabulary for MIRAGE

**File Name:** **Supplementary Data 3**

**Description:** Example of GlycoWorkbench file filled in with structures identified using LC-MS from bovine uterus.
